# The logic of ionic homeostasis: Cations are for voltage, but not for volume

**DOI:** 10.1371/journal.pcbi.1006894

**Published:** 2019-03-14

**Authors:** Andrey V. Dmitriev, Alexander A. Dmitriev, Robert A. Linsenmeier

**Affiliations:** 1 Biomedical Engineering Department, Northwestern University, Evanston, Illinois, United States of America; 2 Independent Researcher, Evanston, Illinois, United States of America; 3 Neurobiology Department, Northwestern University, Evanston, Illinois, United States of America; 4 Ophthalmology Department, Northwestern University, Chicago, Illinois, United States of America; University of Dundee, UNITED KINGDOM

## Abstract

Neuronal activity is associated with transmembrane ionic redistribution, which can lead to an osmotic imbalance. Accordingly, activity-dependent changes of the membrane potential are sometimes accompanied by changes in intracellular and/or extracellular volume. Experimental data that include distributions of ions and volume during neuronal activity are rare and rather inconsistent partly due to the technical difficulty of performing such measurements. However, progress in understanding the interrelations among ions, voltage and volume has been achieved recently by computational modelling, particularly “charge-difference” modelling. In this work a charge-difference computational model was used for further understanding of the specific roles for cations and anions. Our simulations show that without anion conductances the transmembrane movements of cations are always osmotically balanced, regardless of the stoichiometry of the pump or the ratio of Na^+^ and K^+^ conductances. Yet any changes in cation conductance or pump activity are associated with changes of the membrane potential, even when a hypothetically electroneutral pump is used in calculations and K^+^ and Na^+^ conductances are equal. On the other hand, when a Cl^-^ conductance is present, the only way to keep the Cl^-^equilibrium potential in accordance with the changed membrane potential is to adjust cell volume. Importantly, this voltage-evoked Cl^-^-dependent volume change does not affect intracellular cation concentrations or the amount of energy that is necessary to support the system. Taking other factors into consideration (i.e. the presence of internal impermeant poly-anions, the activity of cation-Cl^-^ cotransporters, and the buildup of intra- and extracellular osmolytes, both charged and electroneutral) adds complexity, but does not change the main principles.

## Author summary

We have developed software that calculates membrane potential and cell volume that result from redistribution of principal ions (K^+^, Na^+^, and Cl^-^) during normal cellular activity and experimental manipulations. Calculations in the model are done by an iterative charge-difference method that makes few assumptions about governing equations. Most of the features that were considered to be important for volume and voltage regulation were incorporated in the model, including the unique capability to perform calculations with different values of transmembrane water permeability. We have used the program to reexamine interactions between ionic fluxes, membrane potential, and cell volume and found that there was a previously unappreciated difference in the way that the distribution of cations and anions affect the cell. Na^+^ and K^+^, which are distributed unevenly across the membrane by the Na^+^/K^+^-ATPase, are primarily responsible for the membrane potential, but, contrary to popular belief, do not directly participate in volume regulation. On the other hand, the Cl^-^ conductance determines the extent of volume changes, because Cl^-^ has to follow the changes of membrane potential, which inevitably leads to changes in cell volume. The software is available to download and use for other investigations.

## Introduction

The transmembrane movements of ions during neuronal activity are inevitably associated with changes in ionic concentrations, both intracellularly and extracellularly. This activity-dependent redistribution of ions can be osmotically imbalanced and consequently can lead to changes in volume of the cells and of the extracellular space (ECS). It is usually assumed that three principal ions–Na^+^, K^+^, and Cl^-^—are responsible for the link between transmembrane conductance, voltage and volume alterations associated with neuronal activity. Among them, extracellular K^+^ experiences the largest relative activity-dependent changes. The increase of the extracellular K^+^ concentration ([K^+^]_o_) during neural activity is the easiest to detect, and was recorded first [1, 2], using ion-selective microelectrodes [3]. Soon it was discovered that the increased [K^+^]_o_ induced by electrical stimulation was associated with a 50% reduction of ECS volume ([4] in the cortex of cat). However, it also was shown that the relationship between ions, voltage and volume was not simple, since in some other layers of the cortex, shrinkage of ECS was not detected in spite of considerable elevation of [K^+^]_o_ [4].

The correlation between the increase of [K^+^]_o_ and decrease of ECS volume was found in different parts of the nervous system under various conditions (honey bee eye, light stimulation—[5]; optic nerve, electrical stimulation—[6]; spinal cord, electrical stimulation—[7]; cortex during spreading depression—[8]). However, when extracellular concentrations of Na^+^ and Cl^-^ ([Na^+^]_o_ and [Cl^-^]_o_) were measured to obtain a full picture of the anticipated osmotic imbalance of ions, the results were sometimes confusing. For instance, it was shown that the decrease of ECS volume evoked by electrical stimulation was indeed accompanied by a decrease of [Na^+^]_o_, since Na^+^ enters cells during stimulation, but this decrease had the same amplitude as the [K^+^]_o_ increase, and [Cl^-^]_o_ started to change only after the stimulation, during so called “self-sustained neuronal afterdischarges” [9]. During spreading depression, when ECS was reduced to one fourth of its original volume [8], a large increase of [K^+^]_o_ was indeed exceeded by even larger decrease of [Na^+^]_o_ [10, 11]. But the decrease of [Cl^-^]_o_ was markedly larger (by 12–31 mM) than needed for electrical compensation of extracellular cation deficiency. As discussed further below, these changes still have not been fully explained with a mechanistic model.

The vertebrate retina presents a case of special interest in this respect because it consists of cells that respond to light differently–with predominantly depolarization in proximal layers (ganglion cells, amacrine cells; see S1 Text), but with hyperpolarization in distal layers (photoreceptors, horizontal cells). Accordingly, during illumination the ECS volume decreases in proximal retina, but increases in distal retina [12, 13]. In proximal retina, similarly to brain, ECS shrinkage is associated with a [K^+^]_o_ increase and also with a larger [Na^+^]_o_ decrease and with a compensating [Cl^-^]_o_ decrease [14]. In distal retina the changes are reversed: ECS expansion is associated with a [K^+^]_o_ decrease and with a larger [Na^+^]_o_ increase [14]. However, [Cl^-^]_o_ still decreases in the outer retina when it is expected to increase in order to compensate for total ECS cation excess.

It should be noted that although measuring extracellular ion concentrations with ion-selective microelectrodes is the best available method to obtain data on ionic redistribution during neuronal activity, these measurements are technically difficult, and results must be interpreted with caution. In the case of [Na^+^]_o_ and [Cl^-^]_o_ changes, the measured voltage changes of the ion-selective electrode are small (except in spreading depression) and can be partly compromised by possible electrical artifact arising from a combination of changes in field potential and the huge resistance of the ion-selective microelectrode [14, 15]. Also, the sensors are not absolutely selective; for instance, some Na^+^-selective sensors are influenced by changes in extracellular Ca^2+^ [9], and some Cl^-^-selective sensors respond to pH fluctuations [14]. Most importantly, the changes of extracellular ions and volume evoked by neuronal activity often stimulate reactions of ever present nearby glial elements, which in turn can alter those ionic changes and affect ECS volume [16, 17].

Nevertheless, it is possible to conclude that measurements with ion-selective microelectrodes revealed a certain pattern of related changes in voltage, volume and distribution of the principal ions (K^+^, Na^+^, and Cl^-^) that more or less consistently (within the limits of the method) repeats itself in various parts of the nervous system. Neuronal excitation, which is in most cases associated with depolarization due to an increase of Na^+^ conductance, results in Na^+^ influx responsible for the [Na^+^]_o_ decrease. This Na^+^ influx is electrically compensated partly by outward K^+^ flux and partly by inward Cl^-^ flux, which leads to an increase of [K^+^]_o_ and a decrease of [Cl^-^]_o_. The consequent transmembrane transfer of NaCl into the cells evokes osmotically obliged movement of water, decreases ECS volume, and increases the cell volume. When the neuronal activity is associated with hyperpolarization (like light-induced responses of photoreceptors and horizontal cells in the distal part of the vertebrate retina) the changes of ion concentrations and volumes have opposite signs.

However, the reasons for the voltage-dependent, osmotically imbalanced redistribution of the main ions that lead to volume changes are not entirely clear. Movement of a very small amount of ions is enough for changes of the membrane potential, and that amount has no practical effect on ionic concentration, so ion and volume changes must be more complex than expected from membrane potential considerations alone. The ionic concentrations are changed when the precise balance between influxes and effluxes through membrane passive and active transport systems that existed at rest is temporarily disturbed during activity. For instance, the light-induced decrease of [K^+^]_o_ in the distal retina is a result of a temporal inequity between the passive K^+^ leak out of the photoreceptors, which is quickly reduced by the hyperpolarization, and the active K^+^ pumping into the cell by Na^+^/K^+^-ATPase, which needs time for adjustment [18]. It is natural to assume that the osmotically imbalanced ionic changes that lead to changes in volume could be a result of the unequal exchange of Na^+^ for K^+^ by the Na^+^/K^+^-ATPase (3 Na^+^ out of a cell for 2 K^+^ in). Because of this, the decrease of [Na^+^]_o_ during neuronal depolarization could be expected to be larger than the increase of [K^+^]_o_. That cation imbalance must be electrically compensated by the extracellular decrease in an anion (most probably by Cl^-^) concentration, further diminishing the extracellular osmolarity compared to the intracellular. On the other hand, the passive transmembrane Cl^-^ flux itself is directly affected by changes in membrane potential–attracted into the cell by depolarization and repelled out of the cell by hyperpolarization. In this case, appropriate Na^+^ and/or K^+^ flux is needed for electroneutrality, creating osmotically active NaCl/KCl transfer. Thus, there is currently no simple and single explanation for the link between voltage, volume and ions in the nervous system. Is unequal exchange of Na^+^ for K^+^ by the Na^+^/K^+^-ATPase responsible for osmotically imbalanced redistribution of ions? Or is ion redistribution the consequence of the direct influence of the changing membrane potential on Cl^-^ flux? Maybe both factors play their roles; in this case, what is the contribution of each?

Here we will use computational modeling to provide answers for those questions. Numerous computational models that aim to understand the interrelations among ions, voltage and volume have been developed recently [19–25], (for review see [26, 27]). But most of them are based on modifications of the Goldman equation, and the limitations of this approach (particularly for a dynamic, changing system) have been well described [27]. Alternatively, a much more attractive “charge-difference” method for calculations of simultaneously changing ionic concentrations, membrane potential, and cell volume was introduced [28].

In this work a charge-difference computational model was used for further understanding of the link between ions, voltage, and volume. The list of new and improved features that make our program different from that published earlier is presented in the Methods. The focus was on the specific roles for cations and anions, which is probably the most important feature of the work distinguishing it from current literature, where Na^+^, K^+^, and Cl^-^ were usually treated together. Special attention was directed to the energy requirement to support voltage and volume changes. It also was demonstrated that, contrary to intuitive assumptions, changes of the membrane potential do not necessarily lead to changes in volume, and changes of volume can have no effect on the cation concentration and the membrane potential. Additionally, both Donnan and Double Donnan equilibrium were reexamined. Since the water permeability of the membrane is critically important for Donnan equilibrium, some simulations with various values of this parameter were performed. Our program is the only one that is capable of such calculations.

It should be noted that bicarbonate ion, which is probably the second most important anion after Cl^-^, was omitted here. Although HCO_3_^-^ can move across cell membranes through numerous GABA and glycine channels and by certain cotransporters and exchangers, it is involved in the fundamentally important CO_2_/HCO_3_^-^ buffering system, and its concentration is mostly determined by highly diffusible CO_2_. Accordingly, it is tightly linked to energy metabolism, generation and evacuation of acidic metabolic wastes and other processes that require separate study, and it is beyond the scope of this paper.

The software is offered to share (https://sites.northwestern.edu/ralcomputational/) and significant efforts were directed toward making it flexible and user friendly.

## Methods

### The general description of the model

The model calculates membrane potential (*E*_*m*_) and cell volume (*Vol*) depending on extra- and intracellular concentrations of K^+^, Na^+^, and Cl^-^, their transmembrane conductances, and the activities of the Na^+^/K^+^ ATPase, Na^+^,K^+^,2Cl^-^-cotransporter and K^+^,Cl^-^-cotransporter. These two cation-Cl^-^ cotransporters were included in the model because they directly link Cl^-^ with cations and are prevalent in the nervous system. We also take into account the concentration ([An^-^]_i_) and mean charge valence (z) of intracellular membrane-impermeant osmolytes, which comprise a substantial amount of the intracellular anions. In this respect our model is similar to the most advanced and recent models [23–25, 28]. Additionally, our model has some capabilities which previous models do not have. First, the calculations can be performed with different values of transmembrane water permeability. In existing models on volume regulation very high water permeability is accepted by default, so water instantly follows the ions and other osmolytes, changing the cell volume, but preventing any osmotic difference between the internal and external solutions. In most of our simulations the water permeability was also assumed to be large in order to focus on other aspects of ion-dependent volume-voltage regulation. But in one series of simulations we used the widest possible range of water permeability (from infinity to zero) to investigate its consequences for the cell volume and internal osmolarity. In the extreme case of zero water permeability we can simulate the development of Donnan equilibrium. Second, electrically neutral impermeant osmolytes (like glucose) can be added to extracellular fluid to simulate Double Donnan effects. Third, to simulate certain experimental environments, the model can perform calculations in conditions where osmolyte concentrations change with time. In this part of the work, we modeled the buildup of four different substances: a) external electrically neutral impermeant osmolytes, b) extracellular NaCl, c) internal electrically neutral impermeant osmolytes, and d) internal electrically charged impermeant osmolytes with the addition of an appropriate amount of Na^+^ to maintain electroneutrality. Besides, the model permits various stoichiometries of the Na^+^/K^+^-ATPase, which has been done [24] or can be done [23] in previous work. In most simulations a normal stoichiometry of 3 Na^+^ to 2 K^+^ was used, but we also examined a hypothetical case when an electroneutral pump (3 Na^+^:3 K^+^) was responsible for redistribution of the cations, and some other more exotic stoichiometries were also tested.

With the exception of mentioned above buildups of external NaCl and electrically neutral impermeant osmolytes, all extracellular concentrations during all other simulations were assumed to be constant (as for an isolated cell in a Petri dish).

The parameters are expressed in an easily appreciable physical form and the values are biophysically realistic as far as possible. The conductance of ions is expressed in ions/(sec*V) and can be converted to the usual electrophysiological measure of conductance in Siemens. For instance, the value of 2*10^10^ ions/(sec*V) for total ionic conductance, which was often used in this work, is equal to 3.2*10^−9^ coulombs/(sec*V), i.e. 3.2*10^−9^ Siemens (or 3.2 nS). This corresponds to an input resistance of 312.5 MΩ, a reasonable value for a small sized neuron (for instance, the input resistance of a starburst amacrine cell in the rabbit retina is in the range of 200–250 MΩ [29]. Similarly, the basis for many parameters that were chosen here is experimental work performed mostly on vertebrate retina. For example, a relatively low ratio of gK/gNa is characteristic of the photoreceptors [30, 31], which respond to light with a decrease of gNa [32], as in the Results: “Conductance of Cl^-^ and cell volume.”. The transporter activities are presented in cycles/sec for better comparison to fluxes through the conductances, since the number of cycles/sec is proportional (and in some cases–equal) to the number of ions transferred per second.

### The method of calculation

The same principles for calculations were used here as in the “charge-difference” method of Fraser and Huang [27, 28]. There was no attempt to derive an equation that describes *E*_*m*_ and *Vol* from ionic concentrations and conductances and activities of transporters. Instead, our program 1) counts transmembrane fluxes through the channels and transporters for each ion during a short time period, when (important!) the conditions are assumed to be unchanged, 2) calculates the resulting changes in intracellular ionic concentrations, osmolarity and electrical charge at the end of this time period, and 3) makes appropriate adjustments of the intracellular concentrations, *E*_*m*_ and *Vol*. These three steps in the calculation are described in the sections below (e.g. “The 1^st^ set of calculations”). This cycle repeats over and over again. If a balanced (resting) state was reached, the combined fluxes through all appropriate channels and transporters for each ion (K^+^, Na^+^, and Cl^-^) would equal 0 and ionic concentrations, *E*_*m*_ and *Vol* would not change. When the conditions (a conductance or activity of a transporter) change, the balance will be disturbed, and a new balanced state will be found with potentially new values for *E*_*m*_ and *Vol*, as well as for ionic concentrations. Consequently, at the end of the calculation the activities of the transporters that depend on ionic concentrations can be different from their initial values.

The discretization of a continuous process, which is the core of this method, is an apparent idealization, but it does not affect the precision of results when the resting state is found. And during the dynamic phase, any desired level of accuracy can be achieved by choosing an appropriately short discrete time step. In this work the duration of the time step was 0.1, 0.5, or 1.0 millisecond.

### The 1^st^ set of calculations: The determination of the ionic fluxes

After choosing an initial set of concentrations and pump rates, transmembrane fluxes for each ion (in ions/sec) through each transport system are calculated. Inward fluxes are assumed to have a positive sign, and outward fluxes are considered to be negative, in accordance with the way they affect intracellular ionic concentrations.

#### Passive ionic fluxes through available conductances

To avoid overcomplication, all conductances are assumed to be voltage insensitive and remain constant during calculations. (In real life, this assumption cannot be applied to all neuronal cells, but it is generally correct for vertebrate photoreceptors as well as the majority of other retinal neurons, for retinal pigment epithelium, and for glial cells. We do show (Results: \ **“**Conductance of Cl^-^ and cell volume”**)** what happens when one changes Na^+^ and Cl^-^ conductance and then restores them, and we show how different Cl^-^ conductances influence volume, so it is possible to use the model to study other changes of this type by running the model for a short time, changing conductance, and running it again. However, such changes will not generally result in the steady states we were interested in in this paper.)

$$NaFc=gNa*\left(\left(RT/F\right)*ln\left({\left[Na^{+}\right]}_{o}/{\left[Na^{+}\right]}_{i}\right)‑E_{m}\right);$$ (1)

$$KFc=gK*\left(\left(RT/F\right)*ln\left({\left[K^{+}\right]}_{o}/{\left[K^{+}\right]}_{i}\right)‑E_{m}\right);$$ (2)

$$ClFc=gCl*\left(\left(RT/F\right)*ln\left({\left[Cl^{‑}\right]}_{o}/{\left[Cl^{‑}\right]}_{i}\right)+E_{m}\right);$$ (3)

where, *NaFc*, *KFc*, and *ClFc* are fluxes of Na^+^, K^+^, and Cl^-^, respectively, through their conductances (channels); *gNa*, *gK*, and *gCl* are corresponding conductances; *R* is the universal gas constant; *T* is absolute temperature; *F* is the Faraday constant; and *E*_*m*_ is membrane potential.

Notes: Although the program allows calculations at any temperature, in this work 36.7°C (i.e. 309.85^o^K) was chosen for all calculations. Thus, the equilibrium membrane potential is 61.48 mV for a 10-fold inward-directed concentration gradient of a cation. A negative membrane potential attracts cations into the cell and repels anions from the cell; accordingly, *E*_*m*_ is subtracted for Na^+^ and K^+^, but added in the equation for Cl^-^.

#### Active (ATP-required) fluxes caused by the Na^+^/K^+^-ATPase

$$Ap=Rp/{\left(1+hNa/{\left[Na^{+}\right]}_{i}\right)}^{3};$$ (4)

NaFp=x*(‑1)*Ap; (5)

KFp=y*Ap; (6)

where, *Ap* is the activity of the pump, *Rp* is the rate of the pump, *hNa* is a constant representing the concentration for half-maximal occupation of a Na^+^-binding site on the pump, in all calculations assumed to be 8 mM [33, 34], *NaFp* and *KFp* are fluxes of Na^+^ and K^+^ generated by the Na^+^/K^+^-ATPase, *x* is the number of Na^+^ transferred each cycle (always = 3 in our simulations in Results), *y* is number of K^+^ transferred each cycle (in most cases = 2, but in some calculations = 3, and in one series = 0). Some other values of *x* and *y* were used in simulations mentioned in the Discussion.

Notes: The pump activity (*Ap*) is the actual number of total cycles of all Na^+^/K^+^-ATPase molecules in the cell per second; it is equal to the number of ATP molecules spent per second. The pump activity depends on extra- and intracellular concentrations of Na^+^ and K^+^, as well as ATP concentration. In an energy competent cell the ATP level is stable and the most important regulating factors are [K^+^]_o_ and [Na^+^]_i_. Since in our simulations all ionic extracellular concentrations are constant, only changes in [Na^+^]_i_ are considered to be important. In a restricted range of [Na^+^]_i_ changes it could be enough to assume just a linear relationship between the pump activity and [Na^+^]_i_, as in some previous work [23, 35]. However, Eq 4, similar to the equation that was used by Armstrong [19], more accurately defines the link between [Na^+^]_i_ and the pump activity in our simulations, which deal with both high and low intracellular Na^+^ concentrations. At low Na^+^ concentrations the pump is suppressed, but much more sensitive to changes in [Na^+^]_i_ than at high Na^+^ concentrations. This equation links the pump activity with the pump rate (*Rp*) which is a constant defined by the investigator. *Rp* is proportional to the quantity of Na^+^/K^+^-ATPase molecules in our modeled cell, and represents the theoretical maximum of *Ap* when [Na^+^]_i_ is approaching infinity. *hNa*^*+*^ is analogous to the Michaelis constant *km*, but is the concentration at which *Ap* = *Rp*/8 because the ATPase is a third order reaction in terms of [Na^+^]_i_.

#### Passive fluxes caused by the Na^+^,K^+^,2Cl^—^cotransporter (NKCC)

$$Ankc=Rnkc*log_{10}\left({\left[Na^{+}\right]}_{o}*{\left[K^{+}\right]}_{o}*{\left[Cl^{‑}\right]}_{o}{}^{2}\right)/\left({\left[Na^{+}\right]}_{i}*{\left[K^{+}\right]}_{i}*{\left[Cl^{‑}\right]}_{i}{}^{2}\right));$$ (7)

NaFnkc=KFnkc=Ankc; (8)

ClFnkc=2*Ankc; (9)

where *Ankc* is the NKCC activity, *Rnkc* is the NKCC rate, and *NaFnkc*, *KFnkc*, and *ClFnkc* are fluxes of Na^+^, K^+^, and Cl^-^ generated by the NKCC.

Notes: The cotransporter rate (*Rnkc*) is proportional to the quantity of NKCC molecules in the cell and is constant during the calculation regardless of ionic concentrations (like *Rp* for the Na^+^/K^+^ ATPase). The cotransporter activity (*Ankc*) could be larger or smaller than *Rnkc* depending on the cotransporter driving force, which is expressed by the right part of the multiplication in Eq 7 [36, 37]. Theoretically *Ankc* can also be positive or negative, in contrast to *Rnkc*, which is positive by definition. In normal conditions the product of extracellular concentrations exceeds the product of intracellular concentrations, and as a result *Ankc* is positive, which means the cotransporter moves Na^+^, K^+^, and Cl^-^ into the cell. Also, *Ankc* is equal to the fluxes of Na^+^ and K^+^ and to half of the Cl^-^ flux.

#### Passive fluxes caused by the K^+^,Cl^—^cotransporter (KCC)

$$Akc=Rkc*log_{10}\left({\left[K^{+}\right]}_{o}*{\left[Cl^{‑}\right]}_{o}\right)/\left({\left[K^{+}\right]}_{i}*{\left[Cl^{‑}\right]}_{i}\right));$$ (10)

KFkc=ClFkc=Akc; (11)

where *Akc* is the KCC activity, *Rkc* is the KCC rate, and *KFkc* and *ClFkc* are fluxes of K^+^ and Cl^-^ generated by KCC.

Notes: The cotransporter rate (*Rkc*) is proportional to the quantity of KCC molecules in the cell and is constant during the calculation regardless of ionic concentrations, but the cotransporter activity (*Akc*) depends on extra- and intracellular concentrations of K^+^ and Cl^-^ because they determine the driving forces of the cotransporter [37]. Usually ([K^+^]_o_*[Cl^-^]_o_) < ([K^+^]_i_*[Cl^-^]_i_), so *Akc* is negative. Accordingly, the cotransporter removes K^+^ and Cl^-^ from the cell.

### The 2^nd^ set of calculations: The determination of changes in intracellular ionic concentrations, osmolarity and electrical charge resulting from transmembrane ion transfer

First, fluxes are added separately for each ion and the sums are multiplied by the time step to produce the *amount* of ions that were moved in or out of the cell. Then the amounts are converted into concentrations. Buildups of extra- or intracellular osmolytes defined by the investigator are also taken into account. Buildups are distinguished from fluxes because these are exogenous substances that are added *de novo* to one side of the membrane at a specified rate. They then can affect the concentrations of substances, but never affect electrical charge, since they are either electrically neutral substances or an electrically balanced combination of cations and anions. The concentrations at the beginning of the current time step are marked with index _*b*_ and the concentrations at the end of the time step (yet before possible adjustments for volume changes) are marked with the index _*e*_. To obtain the changes in total intracellular electrical charge, the change in the amount of Cl^-^ is subtracted from the change in the amount of cations and the result is multiplied by the charge of one cation.

dNa=(NaFc+NaFp+NaFnkc)*st; (12)

dK=(KFc+KFp+KFnkc+KFkc)*st; (13)

dCl=(ClFc+ClFpc+ClFnkc+ClFkc)*st; (14)

$${\left[osm\right]}_{o,e}={\left[osm\right]}_{o,b}+bOso*st;$$ (15)

$${\left[Na^{+}\right]}_{o,e}={\left[Na^{+}\right]}_{o,b}+bNaCl*st;$$ (16)

$${\left[Cl^{‑}\right]}_{o,e}={\left[Cl^{‑}\right]}_{o,b}+bNaCl*st;$$ (17)

$${\left[osm\right]}_{i,e}={\left[osm\right]}_{i,b}+bOsi*st;$$ (18)

$${\left[An^{‑}\right]}_{i,e}={\left[An^{‑}\right]}_{i,b}+bAn*st;$$ (19)

$${\left[Na^{+}\right]}_{i,e}={\left[Na^{+}\right]}_{i,b}+dNa/\left(vol_{e}*L\right)+bAn*st*\left(‑z\right);$$ (20)

$${\left[K^{+}\right]}_{i,e}={\left[K^{+}\right]}_{i,b}+dK/\left(vol_{e}*L\right);$$ (21)

$${\left[Cl^{‑}\right]}_{i,e}={\left[Cl^{‑}\right]}_{i,b}+dCl/\left(vol_{e}*L\right);$$ (22)

dQ=(dNa+dK–dCl)*e; (23)

where *dNa*, *dK*, and *dCl* are changes in the intracellular *amount* of respective ions; *st* is the duration of the discrete time step, *[osm]*_*o*_ and *[osm]*_*i*_ are extra- and intracellular concentrations of impermeant neutral osmolytes; *[An*^*-*^*]*_*i*_ is the concentration of internal impermeant anion; *z* is the mean valence of *An*; *bOso*, *bOsi*, *bNaCl*, and *bAn* are “buildups” of extra- and intracellular impermeant neutral osmolytes, external NaCl and of internal impermeant anion, respectively; *vol*_*e*_ is the cell volume at the beginning of the time step; *L* is Avogadro’s number (6.02*10^23^ mol^-1^); *dQ* is the change in internal electrical charge (in coulombs); and *e* is the electrical charge of one cation (1.6*10^−19^ coulomb). “Buildups” are inputs to the model to allow gradual changes in applied concentrations over some period of time.

Notes: All fluxes are added to each other algebraically. For instance, *KFp* and *KFnkc* are positive and increase the amount of intracellular K^+^, but *KFc* and *KFkc* are negative and decrease it (Eq 13). Addition of Cl^-^ (*dCl*) increases the intracellular osmolarity, but it decreases the total intracellular electrical charge; so *dCl* is subtracted from total charge (Eq 23). The buildup of internal impermeant anion (*bAn*) is associated for electroneutrality with buildup of internal Na^+^, which must be multiplied by the mean valence of *An*^*-*^ (-*z*, Eq 20).

### The 3^rd^ set of calculations: The determination of final ionic concentrations, *E*_*m*_ and *Vol* at the end of the time step

The changes in the ionic concentrations obtained in the 2^nd^ set of calculations can affect the intracellular osmolarity. Buildup of extra- or intracellular osmolytes, if defined by the investigator, will result in an additional imbalance in osmolarity. In the 3^rd^ set of calculations, water is allowed to move across the cell membrane to restore osmotic equilibrium by changing the cell volume. The calculations can be performed with different transmembrane water permeability, i.e. with different rates of osmotically driven adjustment of the cell volume. This is an important difference of our program from previous models concerning volume regulation. The change in *E*_*m*_ is found from the change in intracellular charge (*dQ*) divided by the cell membrane capacitance (*c*). The concentrations, *E*_*m*_ and *vol* at the end of the current time step, but before the final volume adjustments, are marked with index _*e*_ and after it with the index _*f*_.

$$E_{m,f}=E_{m,e}+dQ/c;$$ (24)

$$osV=\left({\left[Na^{+}\right]}_{i,e}+{\left[K^{+}\right]}_{i,e}+{\left[Cl^{‑}\right]}_{ie}+{\left[An^{‑}\right]}_{i,e}+{\left[osm\right]}_{i}\right)/\left({\left[Na^{+}\right]}_{o,e}+{\left[K^{+}\right]}_{o,e}+{\left[Cl^{‑}\right]}_{o,e}+{\left[osm\right]}_{o}\right);$$ (25)

VoR=1/tau; (26)

chV=1‑(1‑osV)*VoR*st; (27)

$$vol_{f}=vol_{e}*chV;$$ (28)

$${\left[K^{+}\right]}_{i,f}={\left[K^{+}\right]}_{i,e}/chV;$$ (29)

$${\left[Na^{+}\right]}_{i,f}={\left[Na^{+}\right]}_{i,e}/chV;$$ (30)

$${\left[Cl^{‑}\right]}_{i,f}={\left[Cl^{‑}\right]}_{i,e}/chV;$$ (31)

$$[An^{‑}]{}_{i,f}=[An^{‑}{]}_{i,e}/chV;$$ (32)

$${\left[osm\right]}_{i,f}={\left[osm\right]}_{i,e}/chV;$$ (33)

where *tau* is the time constant of exponential changes of the cell volume in response to a sudden change in osmolarity, *VoR* is a constant inversely related to *tau*, but *VoR* = 1/*st* if *tau* < *st* and *VoR* = 0 if *tau* > 10^8^ sec (more than 3 years), *osV* is a coefficient of osmosis driven volume changes assuming instant transmembrane water movement, *chV* is a coefficient of osmosis driven volume changes corrected for limited water permeability of the membrane.

Notes: The calculations in this part are straightforward, but the section concerning the water permeability and the time constant of volume changes (Eqs 26 and 27) should be explained. Imbalance between intra- and extracellular osmolarity will induce transmembrane movement of water that changes the cell volume and restores osmotic equilibrium. If membrane water permeability is assumed to be infinite, the water shift and volume changes would happen instantly. In reality, the water permeability is high, but not infinite. It is also different in different cell types. The higher the water permeability, the faster the volume change. The water flux, and consequently, the speed of volume changes also depends on the osmotic gradient, and both the flux and the speed decrease with time as the gradient diminishes. This is similar to the well described discharge of a capacitor in a simple RC circuit. In our case the cell volume is analogous to the voltage of the RC circuit, and membrane water resistance (which is the inverse of water permeability)–to the electrical resistance. Thus, the dynamics of cell volume changes evoked by a sudden shift in osmolarity can be described, by analogy with an RC circuit, with the following equation of exponential decay:

$$vol\left(t\right)=vol_{1}+\left(vol_{2}–vol_{1}\right)*\left(1–e^{-t/tau}\right)$$ (34)

where *vol(t)* is the cell volume in time *t*, *vol*_*1*_ is the initial cell volume, *vol*_*2*_ is the final cell volume with osmolarity equilibrated, and *tau* is a time constant, which is inversely proportional to the membrane water permeability. Thus, *tau* is a convenient measure of water permeability, and if water permeability is such that the time constant of the volume change is 1 minute, it means that after 1 minute the cell experiences 63.2% of the expected volume change. Twofold smaller water permeability will correspond to twofold larger time constant of the volume changes, and the cell will need 2 minutes for 63.2% of the volume change.

Eq 25 determines the coefficient *osV*, by which the cell volume and consequently intracellular concentrations must be corrected in order to return to osmotic equilibrium. But due to limited water permeability another coefficient (*chV*), which is a fraction of *osV* and determined by Eq 27, is used later for correction of the cell volume and the intracellular concentrations. The smaller *tau*, the larger *chV* (and closer to *osV*). For practical purposes, in Eq 27 we used *VoR*, which is the inverse of *tau*. It permits a pair of convenient conditions. If *tau* determined by the investigator is 0 or just shorter than the time step (*st*), *VoR* is assumed to be equal to 1/*st*; as a result *osV* = *chV*, i.e. water instantly corrects the cell volume and osmolarity. If extremely low water permeability with *tau* > 10^8^ sec is chosen, *VoR* is assumed to be equal to 0; as a result *osV* = 1, i.e. water does not move across the membrane at all as in simulation of Donnan equilibrium.

After completing the 3^rd^ set of calculations, the program moves forward in time by one time step and repeats all the cycles.

### The modeled cell dimensions and membrane capacitance

**Fig 1:**
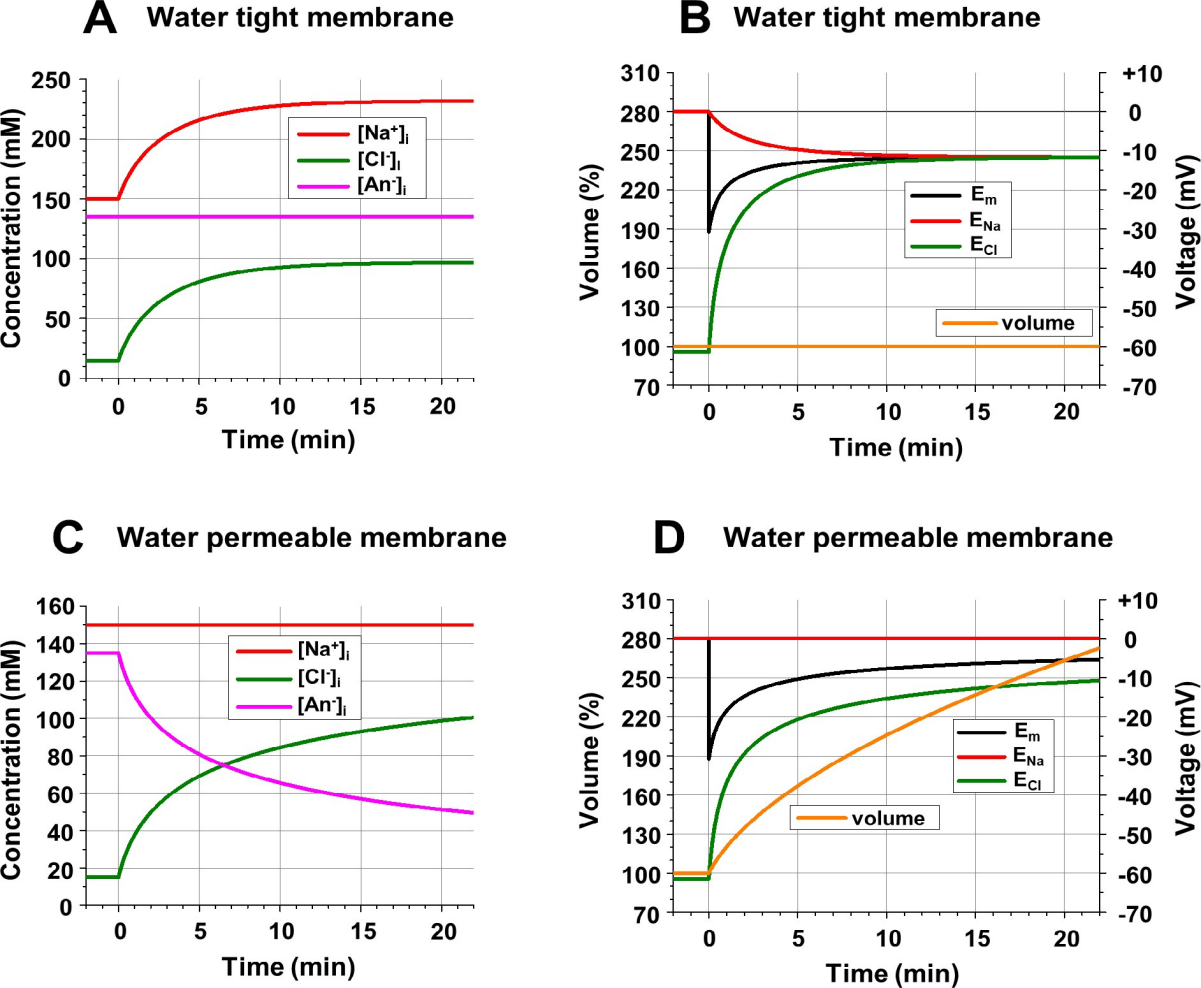
**Intracellular ionic concentrations, membrane potential and cell volume in Donnan (A, B) and non-Donnan (C, D) systems. A**: Changes of [Na^+^]_i_, [Cl^-^]_i_, and impermeable anions ([An^-^]_i_) after opening of *gNa* and *gCl* at time = 0 with no transmembrane transfer of water, which guarantees constant volume and permits a difference in intra- and extracellular osmolarity. **B**: Changes of *E*_*Na*_, *E*_*Cl*_, *E*_*m*_ (scale on the right) associated with ionic changes from the part A; the cell volume is constant (scale on the left, in % of original volume). **C**: Changes of [Na^+^]_i_, [Cl^-^]_i_, and [An-]_i_ after opening of *gNa* and *gCl* with instant osmotically obliged transfer of water, which guarantees equal intra- and extracellular osmolarity, but demands changes in the cell volume. **D**: Changes of *E*_*Na*_, *E*_*Cl*_ and *E*_*m*_ (scale on the right) and of the cell volume (scale on the left, in % of original volume) associated with ionic changes from part C. **Initial conditions:** [Na^+^]_o_ = [Na^+^]_i_ = 150 mM; [Cl^-^]_o_ = 150 mM; [Cl^-^]_i_ = 15 mM; [An^-^]_i_ = 135 mM; z = -1; *gNa* = *gCl* = 0. **Changes at time = 0:** *gNa* = *gCl* = 10^10^ ions/(sec*V).

**Fig 2:**
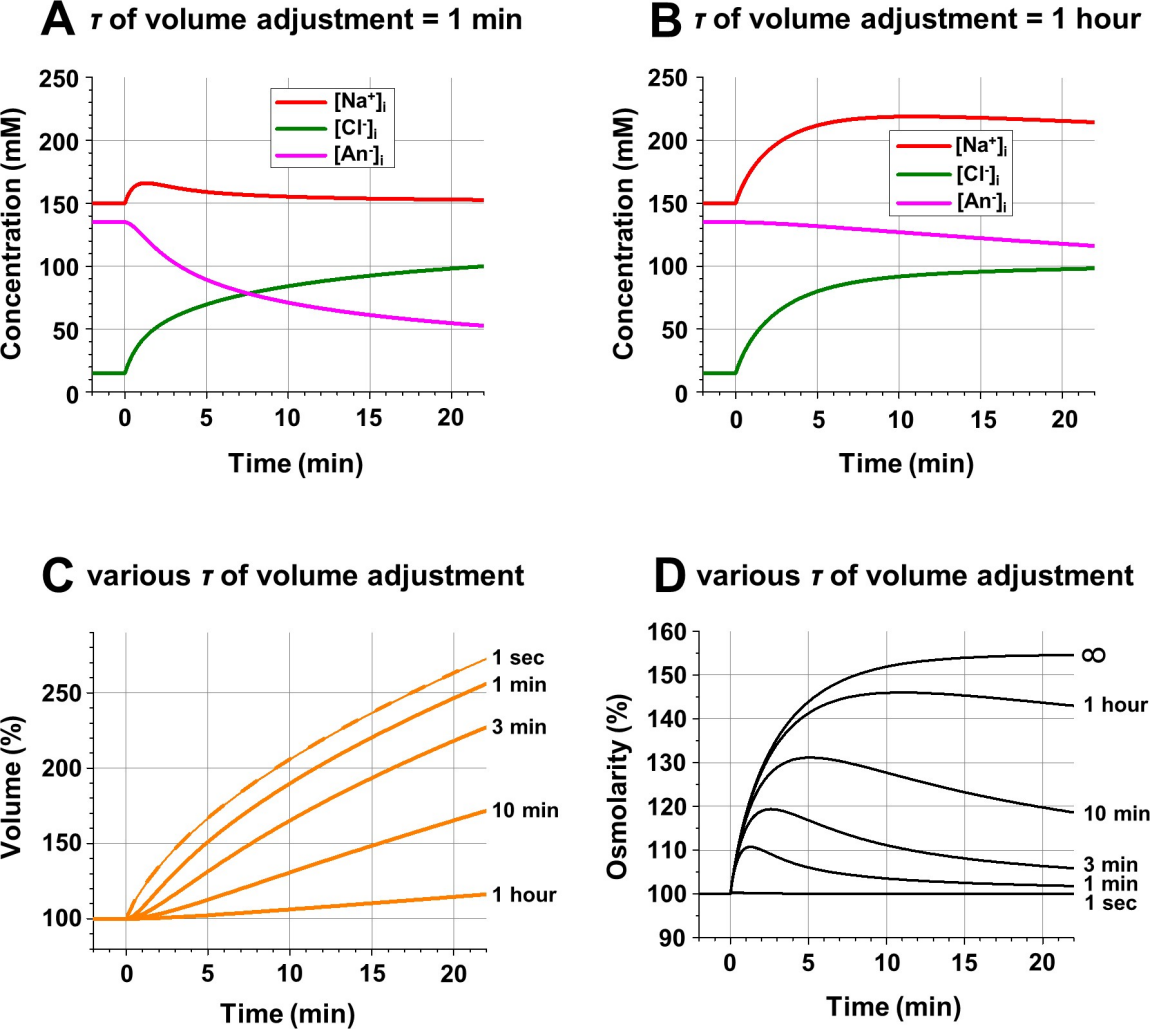
**Intracellular ionic concentrations (A, B), cell volume (C), and intracellular osmolarity (D) at various levels of transmembrane water permeability. A**: Changes of the [Na^+^]_i_, [Cl^-^]_i_, and [An^-^]_i_ after opening of *gNa* and *gCl* when the time constant of water-dependent volume adjustment is 1 minute. **B**: The same when the time constant of water-dependent volume adjustment is 1 hour. **C:** Changes of the cell volume (in % of original volume) at various levels of transmembrane water permeability; to the right of each line—the time constant of water-dependent volume adjustment; the dashed line corresponding to a time constant of 1 second practically overlaps the thin line for the idealization when water-dependent adjustment of cell volume is instantaneous (time constant = 0). **D**: Changes in intracellular osmolarity at various levels of transmembrane water permeability; to the right of each line—the time constant of water-dependent volume adjustment; the time constant of infinity (∞) corresponds to the case when the membrane is not permeable to water. **Initial conditions:** [Na^+^]_o_ = [Na^+^]_i_ = 150 mM; [Cl^-^]_o_ = 150 mM; [Cl^-^]_i_ = 15 mM; [An^-^]_i_ = 135 mM; z = -1; *gNa* = *gCl* = 0. **Changes at time = 0:** *gNa* = *gCl* = 10^10^ ions/(sec*V).

Our modeled cell is assumed to have an effective volume of 7.5*10^−13^ L, equivalent to a cube 10x10x10 μm, with 25% of the volume occupied by organelles (like in rat rod photoreceptors [38]). All calculations are done with respect to this effective cell volume of water containing ions and other osmolytes. Since the surface area of a cube 10x10x10 μm is equal to 6*10^−6^ cm^2^, the total membrane capacitance of the cell is 1.2*10^−11^ F (given a specific capacitance for a neuronal membrane of 2 μF/cm^2^), and remains the same in all calculations. During volume changes cells usually alter their shape but keep the same surface area (for a recent reference see [39]). We specifically pointed out that our model cell is a cube, which permits an increase in cell volume by almost 40% by turning it into a sphere without any changes to surface area, and consequentially with no change in capacitance. During our simulations the volume increases were usually within this range, except in the catastrophic occasion of a swelling cell whose membrane was permeable to Na^+^, Cl^-^ and water, but lacked Na^+^/K^+^-ATPase (Figs 1D and 2C). Nothing prevents the cell from keeping the same surface area when the volume decreases.

**Fig 3:**
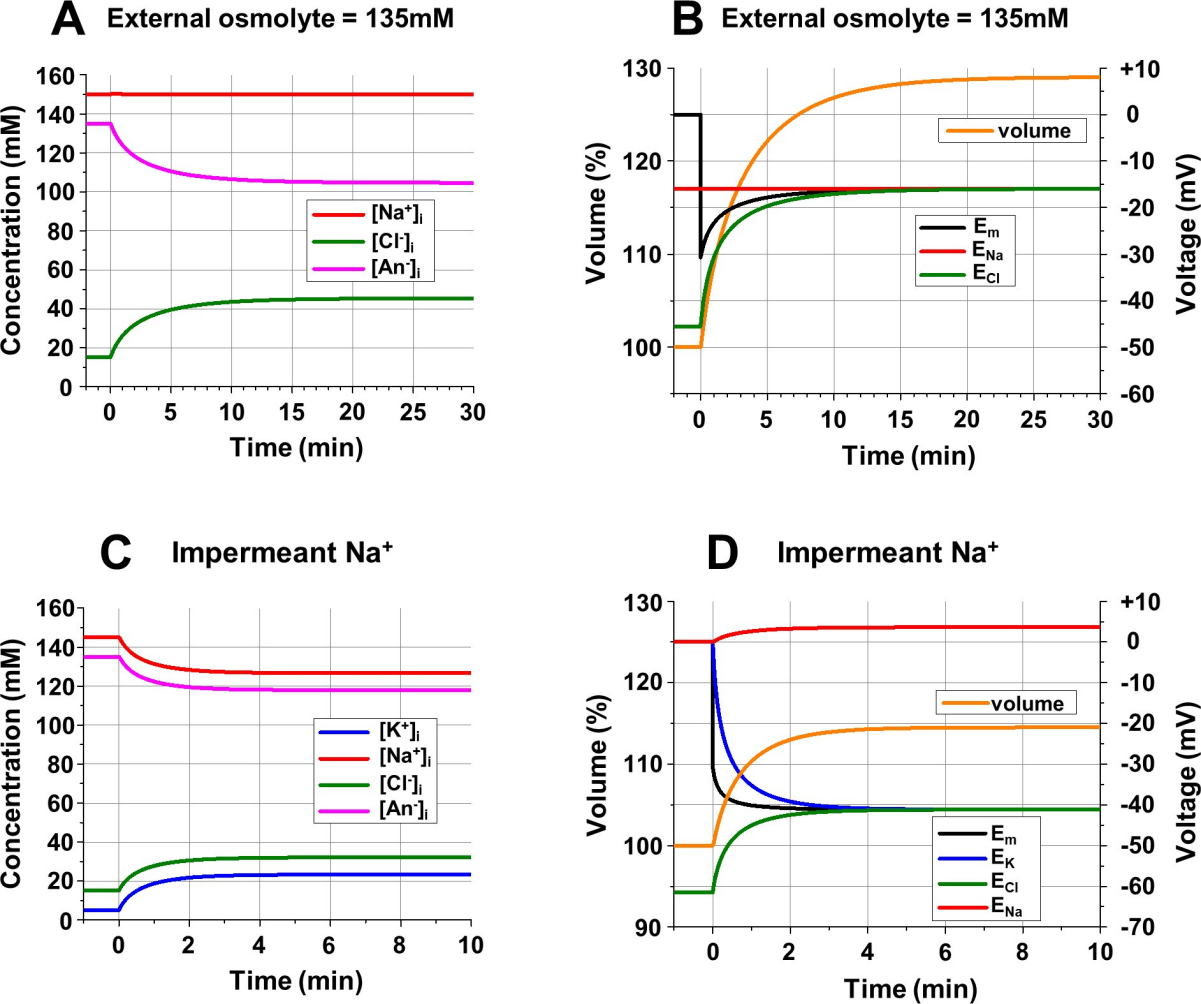
**Intracellular ionic concentrations (A, C), cell volume (B, D), and electrical potentials (B, D) during the development of Double Donnan equilibrium. A**: Changes of [Na^+^]_i_, [Cl^-^]_i_, and [An^-^]_i_ after opening of *gNa* and *gCl* in the presence of a high concentration (135 mM) of an impermeant external neutral osmolyte. **B:** Changes of the cell volume (scale on the left, in % of original volume), and the *E*_*Na*_, *E*_*Cl*_ and *E*_*m*_ (scale on the right) associated with ionic changes in part A. **C**: Changes of the [Na^+^]_i_, [K^+^]_i_, [Cl^-^]_i_, and [An^-^]_i_ after opening of *gK* and *gCl*; the membrane remains impermeable to Na^+^. **D:** Changes of the cell volume (scale on the left, in % of original volume), the *E*_*Na*_, *E*_*K*_, *E*_*Cl*_ and *E*_*m*_ (scale on the right) associated with ionic changes in part C. **Initial conditions (A, B):** [Na^+^]_o_ = 82.5 mM; [Cl^-^]_o_ = 82.5 mM; [neutral osmolyte]_o_ = 135 mM; [Na^+^]_i_ = 150 mM; [Cl^-^]_i_ = 15 mM; [An^-^]_i_ = 135 mM; **(C, D):** [Na^+^]_o_ = [Na^+^]_i_ = 145 mM; [K^+^]_o_ = [K^+^]_i_ = 5 mM; [Cl^-^]_o_ = 150 mM; [Cl^-^]_i_ = 15 mM; [An-]_i_ = 135 mM; (**A-D**): z = -1; *gNa* = *gK* = *gCl* = 0. **Changes at time = 0** (**A, B**): *gNa* = *gCl* = 10^10^ ions/s*V; (**C, D**): *gK* = *gCl* = 10^10^ ions/(sec*V).

**Fig 4:**
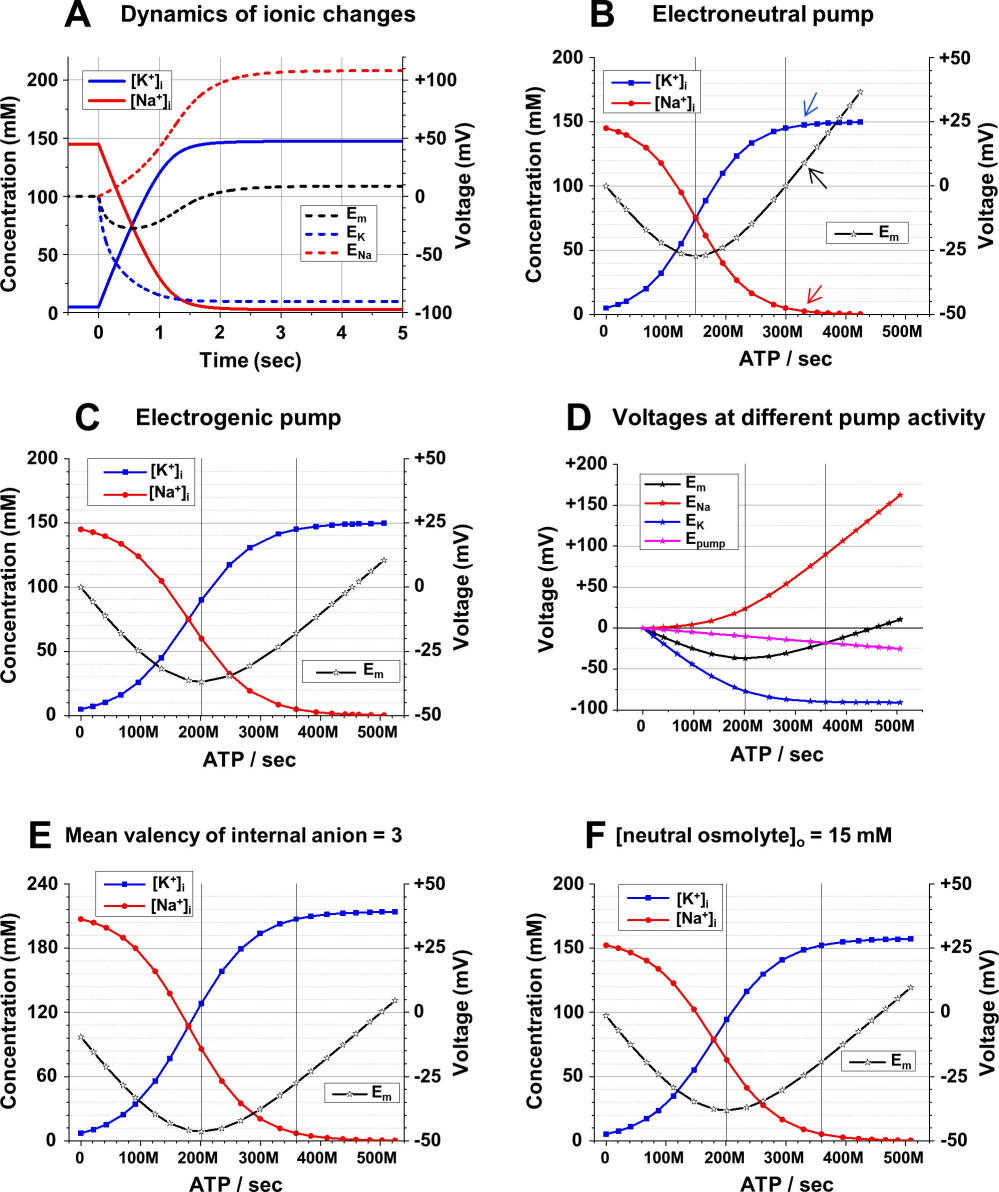
Na^+^/K^+^-ATPase determines intracellular cation concentrations and electrical potentials. **A**: The [Na^+^]_i_, [K^+^]_i_ (scale on the left), *E*_*Na*_, *E*_*K*_, and *E*_*m*_ (scale on the right) during the first 5 seconds after an electrically neutral Na^+^/K^+^ pump was turned on. **B**: Dependence of [Na^+^]_i_, [K^+^]_i_ (scale on the left), and *E*_*m*_ (scale on the right) on steady state activity of the *electrically neutral* Na^+^/K^+^-pump; the left vertical line marks the pump activity when [Na^+^]_i_, = [K^+^]_i_ and *E*_*m*_ was most negative; the right vertical line marks the pump activity when [Na^+^]_i_, and [K^+^]_i_ were reversed from their extracellular values and *E*_*m*_ = 0. Arrows show the values of [Na^+^]_i_, [K^+^]_i_, and *E*_*m*_ under the particular conditions shown in A. **C**: Dependence of [Na^+^]_i_, [K^+^]_i_, (scale on the left), and *E*_*m*_ (scale on the right) on activity of the *electrogenic* Na^+^/K^+^-pump; two vertical lines mark the pump activity when *E*_*m*_ was most negative (but [Na^+^]_i_, ≠ [K^+^]_i_) and when [Na^+^]_i_, and [K^+^]_i_ were reversed from their extracellular values (but *E*_*m*_ ≠ 0). **D**: *E*_*Na*_ and *E*_*K*_ (calculated from [Na^+^]_i_, and [K^+^]_i_, in part C), voltage generated by the electrogenic Na^+^/K^+^-pump (*E*_*pump*_) and analytically calculated final *E*_*m*_ at various activities of the pump. **E**: Dependence of [Na^+^]_i_, [K^+^]_i_, (scale on the left), and *E*_*m*_ (scale on the right) on activity of the electrogenic Na^+^/K^+^-pump when the *mean valence of impermeable anions (z) was = -3*; the vertical lines mark the pump activity when *E*_*m*_ was most negative and when [Na^+^]_i_, and [K^+^]_i_ were reversed. **F**: Dependence of [Na^+^]_i_, [K^+^]_i_, (scale on the left), and *E*_*m*_ (scale on the right) on activity of the electrogenic Na^+^/K^+^-pump when *15 mM of neutral impermeable osmolyte* was added externally; the vertical lines mark the pump activity when *E*_*m*_ was most negative and when [Na^+^]_i_, and [K^+^]_i_ were reversed. **Initial conditions (A—F):** [Na^+^]_o_ = 145 mM; [K^+^]_o_ = 5 mM; [Cl^-^]_o_ = 150 mM; *gNa* = *gK* = 10^10^ ions/(sec*V); **(A—D):** [Na^+^]_i_ = 145 mM; [K^+^]_i_ = 5 mM; [Cl^-^]_i_ = 15 mM; [An^-^]_i_ = 135 mM; z = -1; (**E**): [Na^+^]_i_ = 207.14 mM, [K^+^]_i_ = 7.14 mM, [Cl^-^]_i_ = 21.43 mM and [An^-^]_i_ = 64.29 mM.; z = -3; (**F**): [osm]_o_ = 15 mM; [Na^+^]_i_ = 152.25 mM; [K^+^]_i_ = 5.25 mM; [Cl^-^]_i_ = 15.75 mM; [An^-^]_i_ = 141.75 mM; z = -1; (**A, B**) 3Na^+^/3K^+^ pump; (**C–F**) 3Na^+^/2K^+^ pump. **Changes at time = 0** (**A**): Na^+^/K^+^-ATPase rate = 2.4*10^10^ transfers/sec. (**B-F**) The pump activity presented as ATP molecules spent per second and displayed in engineering notation with *M* for millions.

**Fig 5:**
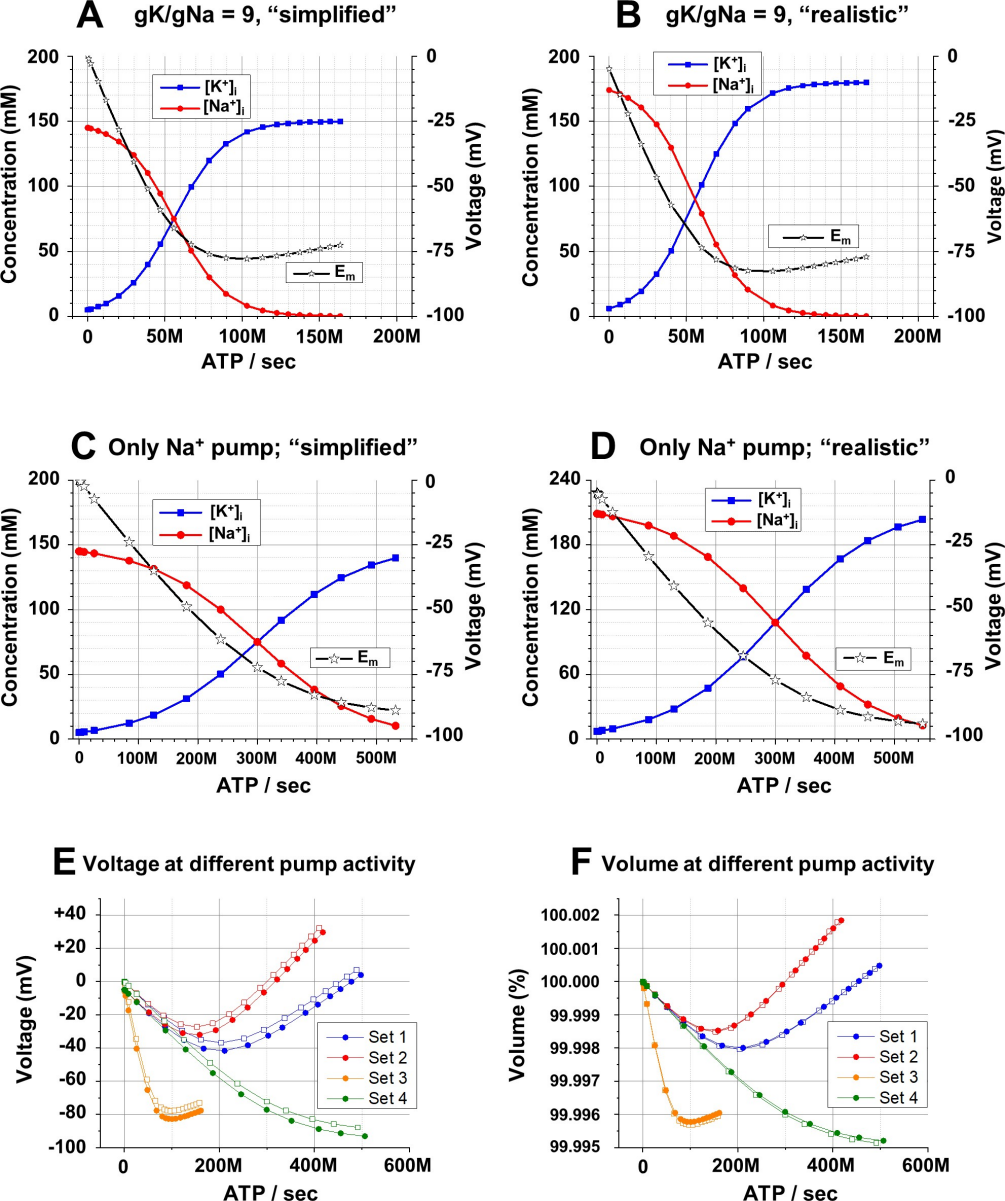
Effects of “simplified” and “realistic” conditions and other parameters on the relation between Na^+^/K^+^-pump activity, *E*_*m*_ and the cell volume. **A**: Dependence of [Na^+^]_i_, [K^+^]_i_, (scale on the left), and *E*_*m*_ (scale on the right) on activity of the electrogenic Na^+^/K^+^-pump *with gK*:*gNa = 9*:*1* in “simplified” (see Methods for explanations) conditions. **B**: The same as in part A, but in “realistic” (see text and Methods for explanations) conditions. **C**: Dependence of [Na^+^]_i_, [K^+^]_i_, (scale on the left), and *E*_*m*_ (scale on the right) on activity of the only Na^+^ pump (3 Na^+^ per ATP) in “simplified” conditions. g*K = gNa*. **D**: The same as in part C, but in “realistic”. **E**: Dependence of *E*_*m*_ on activity of the Na^+^/K^+^-pump activity in both “simplified” (open squares) and “realistic” (closed circles) conditions and under four different settings; the parameters of the settings are described in the text. **F**: Dependence of the cell volume (in % of original volume, i.e. in passive situation with no pumping) on activity of the Na^+^/K^+^-pump in the same conditions and settings as in part E. **Initial conditions (A–D):** [Na^+^]_o_ = 145 mM; [K^+^]_o_ = 5 mM; [Cl^-^]_o_ = 150 mM; **(A, B):** *gNa* = 2*10^9^ ions/(sec*V); *gK* = 1.8*10^10^ ions/s*V; 3Na^+^/2K^+^ pump; **(C, D):** *gNa* = *gK* = 10^10^ ions/s*V; 3Na^+^/0K^+^ pump; **(A, C):** [Na^+^]_i_ = 145 mM; [K^+^]_i_, = 5 mM; [Cl^-^]_i_ = 15 mM; [An^-^]_i_ = 135 mM; z = -1; (**B, D**): [neutral osmolyte]_o_ = 6 mM; [Na^+^]_i_ = 174.0 mM; [K^+^]_i_ = 6.0 mM; [Cl^-^]_i_ = 18.0 mM; [An^-^]_i_ = 108.0 mM; z = -1.5. (**A-F**) The pump activity is presented as ATP molecules spent per second and displayed in engineering notation with *M* for millions.

**Fig 6:**
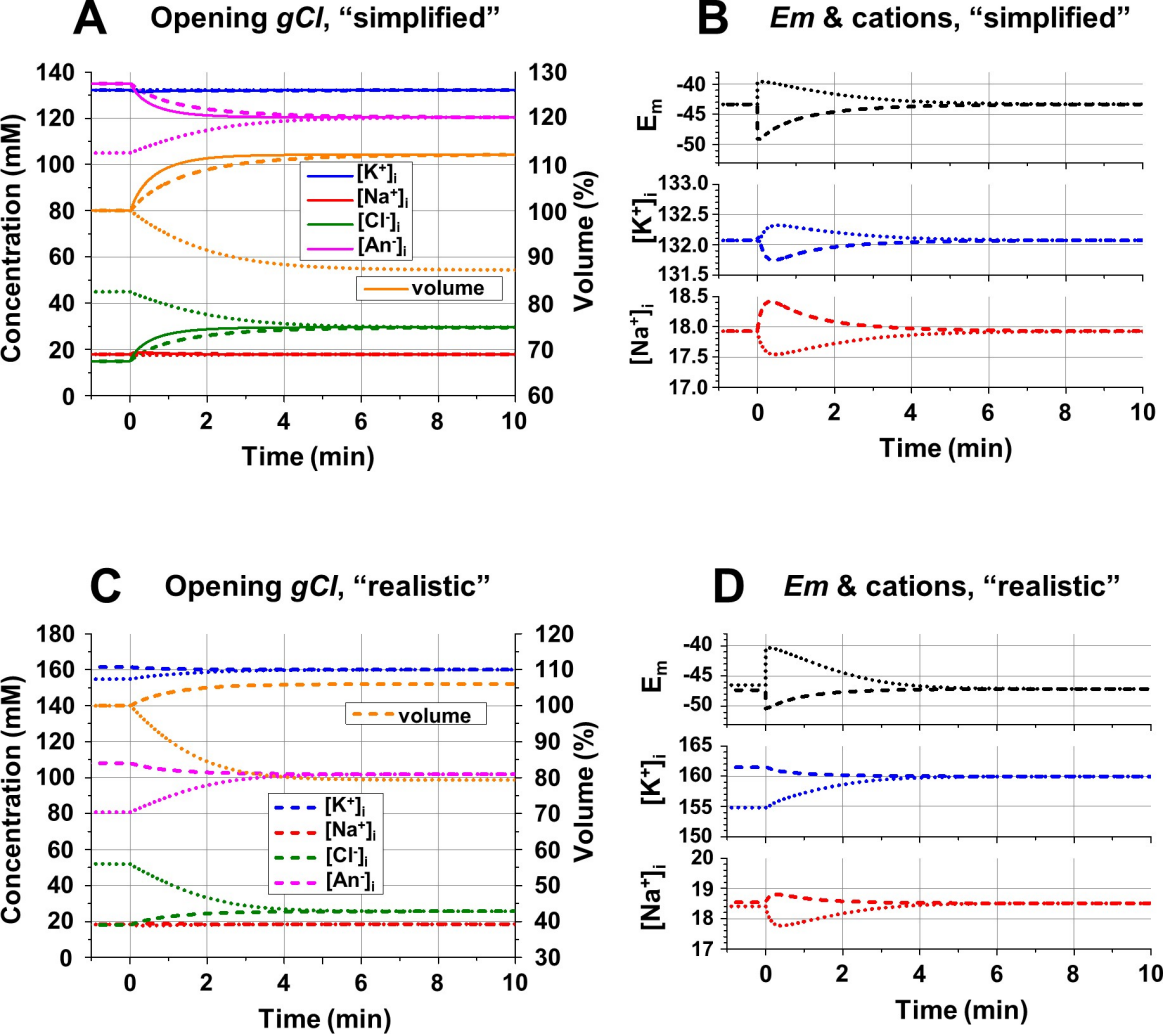
**Effects of opening the Cl**^**-**^ **conductance on ionic concentrations, *E***_***m***_ **and the cell volume in “simplified” (A, B) and “realistic” (C, D) conditions. A**: Changes of the intracellular ion concentrations (scale on the left) and the cell volume (scale on the right, in % of original volume) after opening the Cl^-^ conductance at time = 0; initial [Cl^-^]_i_, = 15 mM (solid and dashed lines) or 45 mM (dotted lines); *gCl* = 10^11^ (solid lines) and 10^10^ ions/(sec*V) (dashed and dotted lines); “simplified” conditions. **B**: Changes of the cation concentrations and *E*_*m*_ after opening the Cl^-^ conductance presented with expanded scale; the lines are as in part A; “simplified” conditions. **C**: Changes of the intracellular ion concentrations (scale on the left) and the cell volume (scale on the right, in % of original volume) after opening the Cl^-^ conductance at time = 0; the initial [Cl^-^]_i_, = 18 mM (i.e. 10% of the total charge of intracellular anions—solid and dashed lines) or 52 mM (i.e. 30% of the total charge of intracellular anions—dotted lines); *gCl* = 10^10^ ions/(sec*V); “realistic” conditions. **D**: Changes of the cation concentrations and *E*_*m*_ after opening the Cl^-^ conductance presented with expanded scale; the lines are as in part C; “realistic” conditions. **Initial conditions (A—D):** [Na^+^]_o_ = 145 mM; [K^+^]_o_ = 5 mM; [Cl^-^]_o_ = 150 mM; *gNa* = 8*10^9^ ions/(sec*V); *gK* = 1.2*10^10^ ions/(sec*V); 3Na^+^/2K^+^ pump with activity of 2.64*10^8^ ATP/sec; **(A, B):** [Na^+^]_i_ = 17.9 mM; [K^+^]_i_, = 132.1 mM; [Cl^-^]_i_ = 15 or 45 mM; [An^-^]_i_ = 135 or 105 mM; z = -1; (**C, D**): [neutral osmolyte]_o_ = 6 mM; [Na^+^]_i_ = 18.5 mM; [K^+^]_i_ = 161.5 mM; [Cl^-^]_i_ = 18.0 mM; [An^-^]_i_ = 108.0 mM (if Cl^-^ contributes 10% of negative charge) or [Na^+^]_i_ = 18.4 mM; [K^+^]_i_ = 154. 8 mM; [Cl^-^]_i_ = 52.0 mM; [An^-^]_i_ = 80.8 mM (if Cl^-^ contributes 30% of negative charge); z = -1.5. **Changes at time = 0** (**A**): *gCl* = 10^11^ or 10^10^ ions/(sec*V); (**B**—**D**): *gCl* = 10^10^ ions/(sec*V).

**Fig 7:**
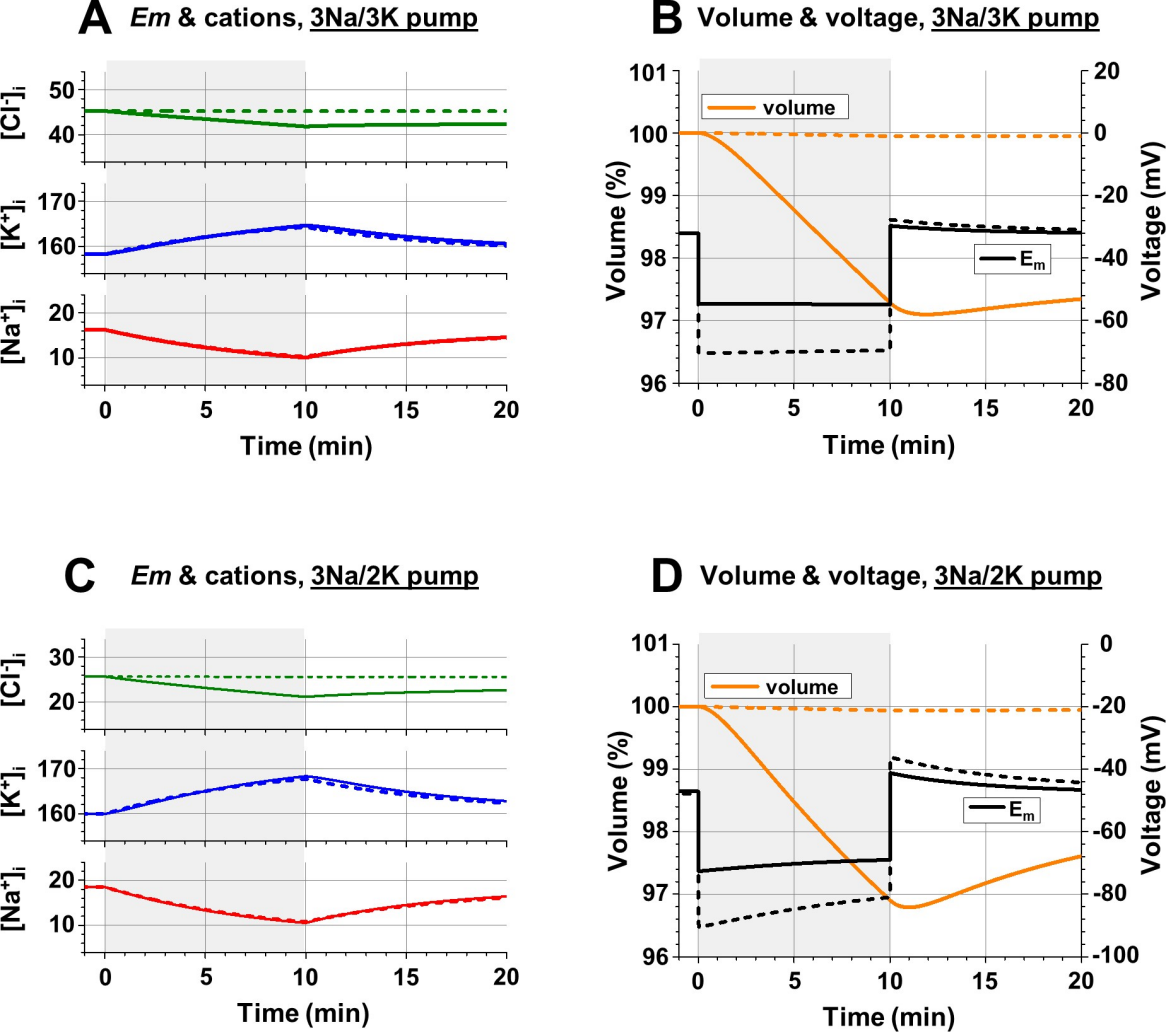
**Changes of intracellular ionic concentrations (A, C), *E***_***m***_ **and the cell volume (B, D) evoked by temporary reduction of Na**^**+**^ **conductance. A**: Changes of [Na^+^]_i_, [K^+^]_i_, and [Cl^-^]_i_, after reduction of *gNa* to 2*10^9^ ions/(sec*V) for 10 seconds starting at time = 0 (grey area) and then returning to the original *gNa* of 8*10^9^ ions/(sec*V) for the next 10 seconds; *gCl* = 10^10^ (solid lines) and 1*10^8^ ions/(sec*V) (dashed lines); imaginary electroneutral 3Na^+^/3K^+^-pump, activity of 2.41*10^8^ ATP/sec. **B**: Changes of the cell volume (scale on the left, in % of original volume) and voltage (scale on the right) with the same temporary reduction of *gNa*; the lines are as in part A. **C**: and **D**: the same as parts A and B, respectively, but with an electrogenic 3Na^+^/2K^+^-pump, activity 2.72*10^8^ ATP/sec. All simulations were in “realistic” conditions. **Initial conditions (A-D):** [Na^+^]_o_ = 145 mM; [K^+^]_o_ = 5 mM; [Cl^-^]_o_ = 150 mM; [neutral osmolyte]_o_ = 6 mM; *gNa* = 8*10^9^ ions/(sec*V); *gK* = 1.2*10^10^ ions/(sec*V); **(A, B):** [Na^+^]_i_ = 16.3 mM; [K^+^]_i_, = 158.3 mM; [Cl^-^]_i_ = 45.3 mM; [An^-^]_i_ = 86.2 mM; z = -1.5; 3Na^+^/3K^+^ pump; (**C, D**): [Na^+^]_i_ = 18.5 mM; [K^+^]_i_ = 160.0 mM; [Cl^-^]_i_ = 25.7 mM; [An^-^]_i_ = 101.9 mM; z = -1.5; 3Na^+^/2K^+^ pump. **Changes at time = 0** (**A-D**): *gNa* = 2*10^9^ ions/(sec*V); **Changes at time = 10 sec** (**A-D**): *gNa* = 8*10^9^ ions/(sec*V).

**Fig 8:**
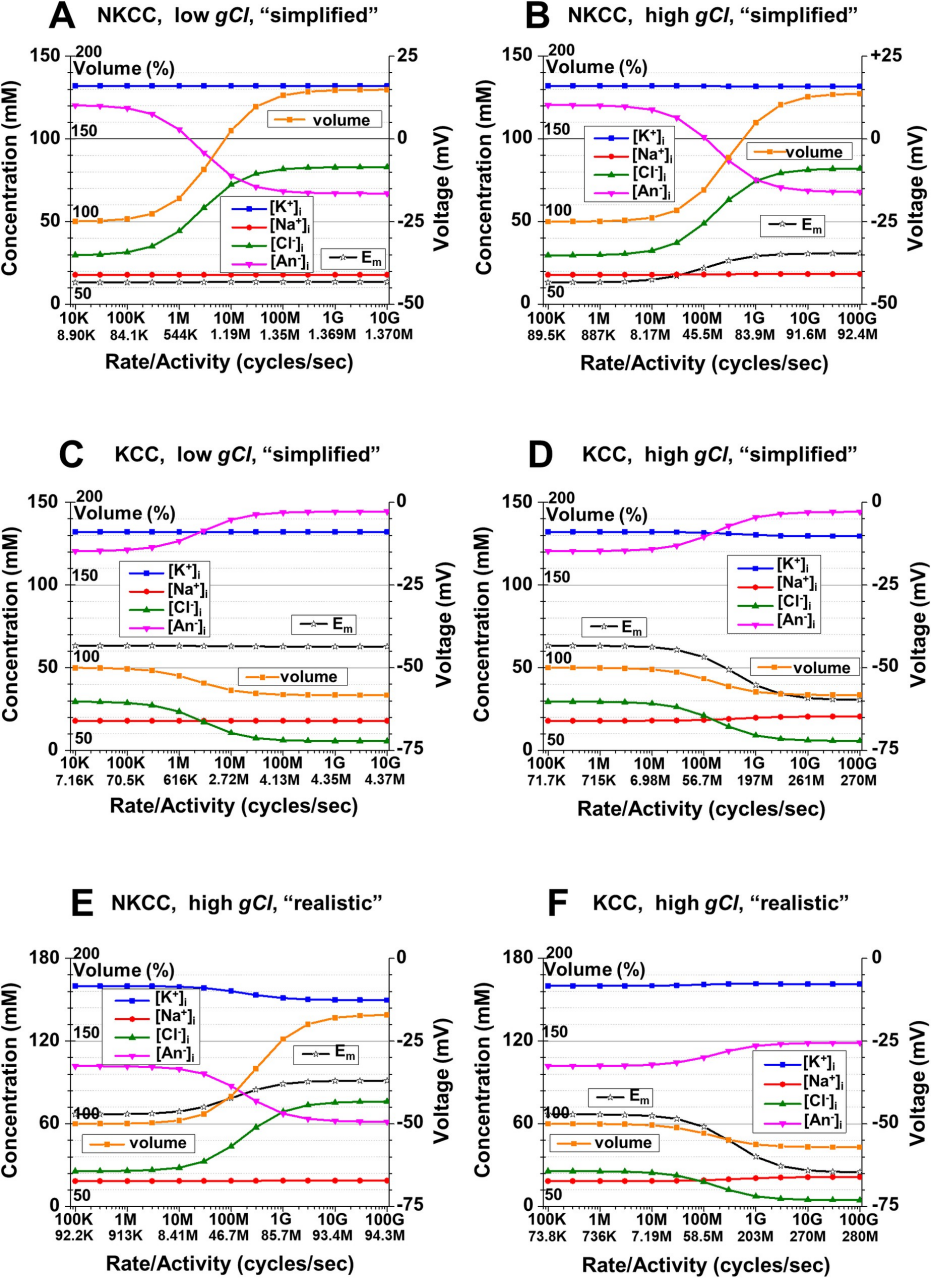
The intracellular ionic concentrations, *E*_*m*_ and the cell volume as a function of the activity of cation-Cl^-^ cotransporters. **A**: [Na^+^]_i_, [K^+^]_i_, [Cl^-^]_i_, and [An^-^]_i_, (left axis, with labels to the left of the axis), the cell volume (left axis, with labels to the right of the axis, in % of original volume, i.e. with no cotransporter activity), and *E*_*m*_ (scale on the right) as a function of Na^+^,K^+^,2Cl^-^ cotransporter rate and corresponding activity at steady state (lower row of labels) in cycles/sec displayed in engineering notation with *K* for thousands, *M* for millions and *G* for billions; low *gCl* (10^8^ ions/(sec*V)). **B**: The same as part A, but with high *gCl* (10^10^ ions/(sec*V)). **C**: The ionic concentrations, cell volume, and *E*_*m*_ as a function of K^+^,Cl^-^ cotransporter activity; low *gCl* (10^8^ ions/(sec*V)). **D**: The same as part C, but with high *gCl* (10^10^ ions/(sec*V)). All simulations in parts A -D were in “simplified” conditions. **E**: The same as part B, but in “realistic” conditions. **F**: The same as part D, but in “realistic” conditions. **Initial conditions (A-F):** [Na^+^]_o_ = 145 mM; [K^+^]_o_ = 5 mM; [Cl^-^]_o_ = 150 mM; *gNa* = 8*10^9^ ions/(sec*V); *gK* = 1.2*10^10^ ions/(sec*V); 3Na^+^/2K^+^ pump, activity 2.64*10^8^ ATP/sec; **(A-D):** [Na^+^]_i_ = 17.9 mM; [K^+^]_i_, = 132.1 mM; [Cl^-^]_i_ = 29.6 mM; [An^-^]_i_ = 120.4 mM; z = -1; (**E, F**): [neutral osmolyte]_o_ = 6 mM; [Na^+^]_i_ = 18.5 mM; [K^+^]_i_ = 160.0 mM; [Cl^-^]_i_ = 25.7 mM; [An^-^]_i_ = 101.9 mM; z = -1.5.

**Fig 9:**
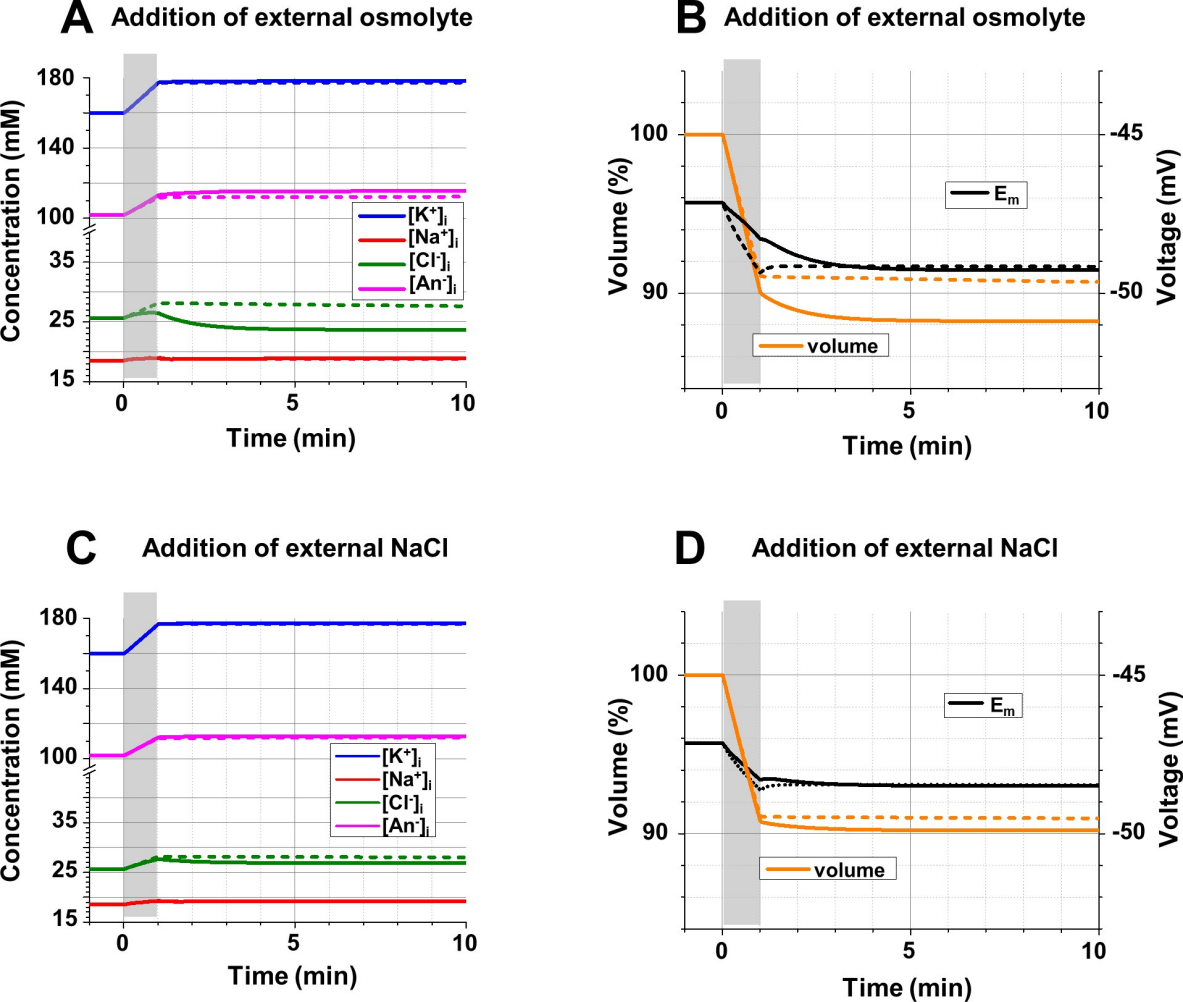
**The intracellular ionic concentrations (A, C), *E***_***m***_ **and the cell volume (B, D) during and after buildup of external osmolytes. A**: Changes of [Na^+^]_i_, [K^+^]_i_, [Cl^-^]_i_, and [An^-^]_i_ resulting from an increase of the concentration of an external impermeable neutral osmolyte by 0.5 mM/sec during 1 min (gray area); *gCl* = 10^10^ ions/(sec*V) (solid lines) and 10^8^ ions/(sec*V) (dashed lines); note the different scale before and after the break in the Y-axis (concentrations). **B**: Changes of the cell volume (scale on the left, in % of original volume), and *E*_*m*_ (scale on the right) accompanying the concentration changes in part A; lines as in part A. **C**: Changes of [Na^+^]_i_, [K^+^]_i_, [Cl^-^]_i_, and [An^-^]_i_ resulting from an increase of [Na]_o_ and [Cl]_o_ by 0.25 mM/sec during 1 min (gray area); lines as in part A; note the different scale before and after the break in the Y-axis (concentrations). **D**: Changes of the cell volume (scale on the left, in % of original volume), and *E*_*m*_ (scale on the right) accompanying the concentration changes in part C; lines as in part A. **Initial conditions (A-D):** [Na^+^]_o_ = 145 mM; [K^+^]_o_ = 5 mM; [Cl^-^]_o_ = 150 mM; [neutral osmolyte]_o_ = 6 mM; *gNa* = 8*10^9^ ions/(sec*V); *gK* = 1.2*10^10^ ions/(sec*V); 3Na^+^/2K^+^ pump, activity 2.72*10^8^ ATP/sec; [Na^+^]_i_ = 18.5 mM; [K^+^]_i_ = 160.0 mM; [Cl^-^]_i_ = 25.7 mM; [An^-^]_i_ = 101.9 mM; z = -1.5; all—“realistic” conditions.

**Fig 10:**
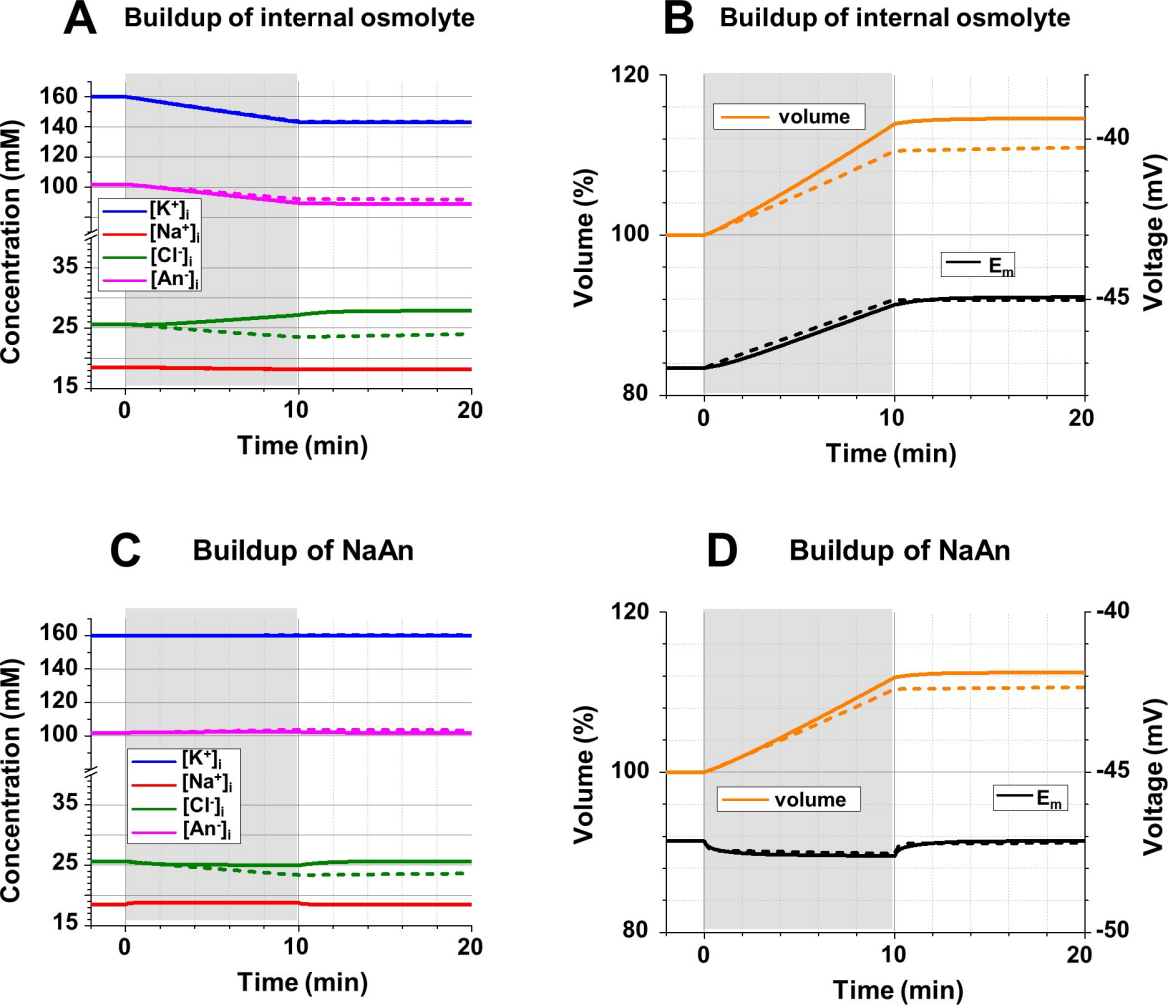
**The intracellular ionic concentrations (A, C), *E***_***m***_ **and the cell volume (B, D) during and after buildup of internal osmolytes. A**: Changes of [Na^+^]_i_, [K^+^]_i_, [Cl^-^]_i_, and [An^-^]_i_ resulting from an increase of the concentration of an internal external impermeable neutral osmolyte by 0.05 mM/sec during 10 min (gray area); *gCl* = 10^10^ ions/(sec*V) (solid lines) and 10^8^ ions/(sec*V) (dashed lines). **B**: Changes of the cell volume (scale on the left, in % of original volume), and *E*_*m*_ (scale on the right) accompanying the concentration changes in part A; lines as in part A. **C**: Changes of [Na^+^]_i_, [K^+^]_i_, [Cl^-^]_i_, and [An^-^]_i_ resulting from an increase of the concentration of AnNa_x_ by 0.02 mM during 10 min (gray area); x = -z = 1.5; and lines as in part A; note the different scale before and after the break in the Y-axis (concentrations). **D**: Changes of the cell volume (scale on the left, in % of original volume), and *E*_*m*_ (scale on the right) accompanying the concentration changes in part C; lines as in part A. **Initial conditions (A-D):** [Na^+^]_o_ = 145 mM; [K^+^]_o_ = 5 mM; [Cl^-^]_o_ = 150 mM; *gNa* = 8*10^9^ ions/(sec*V); [neutral osmolyte]_o_ = 6 mM; *gK* = 1.2*10^10^ ions/(sec*V); 3Na^+^/2K^+^ pump, activity 2.72*10^8^ ATP/sec; [Na^+^]_i_ = 18.5 mM; [K^+^]_i_ = 160.0 mM; [Cl^-^]_i_ = 25.7 mM; [An^-^]_i_ = 101.9 mM; z = -1.5; all—“realistic” conditions.

The initial conditions and manipulations of the examined system (concentrations, conductances, transporter activities) varied from simulation to simulation, and their exact values are listed in the figure legends. It should be noted that initial conditions that describe the starting point of the system before manipulations can refer to equilibrium states (Figs 1–5) or nonequilibrium but steady states with stable concentrations and *Em* (Figs 6–10). In the second case ionic conductances and the rate of the Na^+^/K^+^-ATPase were chosen for reasons explained in the text, but concentrations and *Em* that are given as initial conditions in the figure legends were the results of preparatory calculations that led to the state of the system at t = 0. Preparatory calculations were also necessary to determine initial conditions in some equilibrium states. One particular set of conditions includes 6 mOsm external impermeant osmolyte and a valence of -1.5 for internal impermeant anions, which exemplifies an osmolarity-charge asymmetry, i.e. conditions when the quantity of equivalent charge is not the same as the quantity of osmotically active molecules. Since such asymmetry is expected to be common in real cells, this set is called the “realistic” conditions and often will be compared to “simplified” conditions, which are symmetrical in osmolarity-charge respect, when the valence of internal impermeant anions is equal to -1 and there are no other external osmolytes besides NaCl and KCl.

## Results

The aim of this work is to examine the interactions between ionic concentrations, membrane potential, and cell volume in a complex system containing most of the components which are considered to be important for the matter. However, in order to determine the specific roles of those components, it is convenient to dissect the system into simpler subsystems. Accordingly, we will first look at systems based on Donnan (Figs 1 and 2) and Double Donnan (Fig 3) equilibrium which can keep the membrane potential stable (and different from 0) without energy expenditure. Then we will focus on a system in which the membrane is permeable only to cations (Na^+^ and K^+^) and contains the Na^+^/K^+^-ATPase, with the additional complexity of an uneven transmembrane distribution of neutral osmolytes and a deviation of the mean valence of the impermeant internal anions from -1 (Figs 4 and 5). The Cl^-^ conductance (*gCl*) and cation-Cl^-^ cotransporters (NKCC and KCC) will be added in the third group of simulations (Figs 6, 7 and 8). Finally, we will examine the changes in cell volume and the membrane potential which are associated with the buildup of osmolytes, internal and external, electrically neutral and charged, permeant and impermeant, that roughly simulate regulatory volume increases (RVI) (Figs 9 and 10).

### Donnan equilibrium or how to get a membrane potential for free

The classical example of a system that generates a considerable transmembrane potential without spending energy is the one based on Donnan equilibrium. The conditions which can lead to Donnan equilibrium—unequal distribution of Cl^-^ across the cell membrane and selective permeability of the membrane to cation(s) and to Cl^-^, but not to other intracellular anions–are typical for living cells, including neurons. Those impermeant “other intracellular anions” consist of a diverse group of large and small molecules which contribute noticeably to the voltage-volume regulation, and they are addressed specifically later. For the current simulation of Donnan equilibrium let us just assume that the impermeant anions ([An^-^]_i_) account for most of the internal anion concentration (135 mM) and have a mean valence = -1. At the beginning (before time = 0) the membrane is permeable to nothing, the membrane potential is 0 mV, and for the osmotic and electrical balance the extracellular Cl^-^ concentration ([Cl^-^]_o_) is 150 mM, the intracellular Cl^-^ concentration ([Cl^-^]_i_) is 15 mM, and cation concentrations (in this case Na^+^) are 150 mM, both inside and outside. The membrane remains impermeable to water, so the cell volume does not change.

The results of the simulations are presented in Fig 1A and 1B. At time = 0 the cell begins to be permeable to Cl^-^ and to Na^+^. In the simulation, *gNa* and *gCl* are set to be equal. Cl^-^ immediately starts to move into the cell because [Cl^-^]_o_ is 10 times larger than [Cl^-^]_i_. Rapidly the influx of Cl^-^ brings negative charge into the cell and hyperpolarizes the membrane. This creates an electrical driving force for Na^+^ influx, and the entering Na^+^ partly neutralizes intracellular negativity. As a result, Cl^-^ continues to enter because its strong inward concentration-dependent driving force exceeds its outward electrical driving force. Na^+^ also continues to enter because its inward electrical driving force exceeds its weak outward concentration-dependent driving force. In 20 minutes both [Na^+^]_i_ and [Cl^-^]_i_ are increased by 81.8 mM and practically stabilized. At these concentrations (231.8 mM for [Na^+^]_i_ and 96.8 mM for [Cl^-^]_i_) their Nernst potentials are both equal to the membrane potential *E*_*m*_ = - 11.6 mV (Fig 1B), which means that the concentration-driven fluxes are counter-balanced by electrically-driven fluxes for both ions and the system has reached a stable equilibrium. This is Donnan equilibrium, and as expected [Na^+^]_i_ * [Cl^-^]_i_ = [Na^+^]_o_ * [Cl^-^]_o._ Indeed, 231.8 * 96.8 = 22438.24 ≈ 22500 = 150 * 150. Additional time is required to achieve a more accurate fit between the results of the calculation and the Donnan expectations. With a chosen accuracy of 15 significant digits, the concentrations are finally stabilized after about 90 min at 231.986477689649 mM for [Na^+^]_i_ and 96.9884116601443 mM for [Cl^-^]_i_; their product is 22499.9999977506.

Special calculations were not performed for validating the model but results like these (as well as some others that will be presented later) demonstrate the model’s validity. It is also worth mentioning that the initial change of *E*_*m*_, which looks instantaneous in Fig 1B, in reality decreases exponentially with a time constant of 3.7 ms (see S1 Fig), and this is exactly what is expected in our cell, which was set to have an input resistance of 312.5 MΩ and a membrane capacitance of 12 pF.

Increasing both [Na^+^]_i_ and [Cl^-^]_i_, of course, increases the total intracellular osmolarity. In this example the intracellular osmolarity increased from 300 to 463.6 mOsm ([Na^+^]_i_ = 231.8 mM, [Cl^-^]_i_ = 96.8 mM, and [An^-^]_i_ = 135 mM), creating a strong osmotic gradient. Thus, another fundamental condition that is necessary for Donnan equilibrium is the prevention of transmembrane movement of osmotically obliged water. Otherwise the water enters the cell following ions, increasing the cell volume and diluting ionic concentrations. Exactly that happened when the same simulations as for Fig 1A and 1B were repeated, but under the assumption that the membrane was highly water-permeable, and water instantly compensated for the potential osmotic imbalance associated with ionic transfer (Fig 1C and 1D). As in the previous simulation, opening of Cl^-^ and Na^+^ conductances permits both ions to enter the cell (Cl^-^ due to the concentration gradient, and Na^+^ because of the intracellular negativity created by the influx of Cl^-^). But water also enters, increasing cell volume and diluting intracellular concentrations. One of the effects of the dilution is a decrease of the impermeant anion concentration [An^-^]_i_. The other is that the [Na^+^]_i_ remains the same and equal to [Na^+^]_o_ because the increase in the amount of Na^+^ is precisely compensated by the increase in volume. As a result, the equilibrium potential for Na^+^ is 0, and Na^+^ will continue to enter the cell as long as the membrane potential stays negative. Cl^-^ also will continue to enter (and [Cl^-^]_i_ will continue to increase in spite of the dilution) because its equilibrium potential is twice as negative as the membrane potential. Speaking of the membrane potential, in a system of two unevenly distributed ions with different equilibrium potentials, the *E*_*m*_ will obviously be somewhere in between those potentials. In this case, when the membrane is equally permeable to both Cl^-^ and Na^+^, *E*_*m*_ is the arithmetic mean of their Nernst potentials: *E*_*m*_ = (*E*_*Cl*_ + *E*_*Na*_) / 2. Since *E*_*Na*_ = 0, *E*_*m*_ should be equal to half of *E*_*Cl*_. Again, the simulations show exactly what is theoretically expected.

Fig 1C and 1D show that the addition of water permeability to a system which consists of permeable Na^+^ and Cl^-^ and impermeant An^-^ makes equilibrium unattainable. The equilibrium requires that *E*_*m*_ = *E*_*Na*_ = *E*_*Cl*_, and since *E*_*Na*_ = 0 it is only possible if [Cl^-^]_i_ = [Cl^-^]_o_ and accordingly [An^-^]_i_ = 0. It should be remembered that in the simulation of Fig 1C and 1D the water permeability was assumed to be extremely large, permitting no osmotic imbalance. Although this idealization is not too far from reality and is usually accepted as true in computational models of volume regulation, it is still unrealistic. So Fig 2 shows a series of simulations with the same initial conditions as for Fig 1 but using various values of water permeability expressed for simplicity as a time constant of osmotic volume adjustment (see Methods). At a reasonable water permeability (time constant = 1 min, Fig 2A) the changes of ionic concentrations were similar to the data presented in Fig 1C (instant transmembrane water transfer), and at very low water permeability (time constant = 1 hour, Fig 2B) the concentrations were similar to the data presented in Fig 1A (no transmembrane water transfer at all). The larger the water permeability (shorter time constant), the faster the volume increase (Fig 2C). At a time constant of 1 sec the volume increase is indistinguishable from that under the assumption of instant water movement, and it is not much different at a realistic time constant of 1 min. Importantly, even with unrealistically low water permeability (time constant = 1 hour) the cell volume will increase slowly but steadily, theoretically, to infinity, and practically until the cell blows up.

Also, the smaller the water permeability (longer time constant), the larger the transmembrane osmotic gradient (Fig 2D). Bacterial and plant cells can counteract the osmotic pressure with hydrostatic pressure because they have rigid cell walls to preserve the cell volume. Animal cells have no such walls, and their ability to withstand osmotic pressure is limited. Thus, in animals, osmotically imbalanced transmembrane transfer of ions is inevitably associated with changes in cell volume. The time constant of volume adjustment of neuronal and glial cells is in the range of tens of seconds to a minute. For instance, the osmotically evoked volume increase of retinal Muller cells has a time constant of 0.5–1.0 minute [40]. The light-induced volume changes of vertebrate photoreceptors have approximately the same dynamics, judging by changes of ECS in the retina [13, 14].

For all other calculations in this work, simulations were done with a highly water permeable membrane (volume time constant = 1 sec) in order to focus on other aspects of voltage-volume regulation. This will affect the dynamics (although slightly, as indicated by the similarity of the 1 min and 1 sec curves in Fig 2C), but not the overall conclusions.

As we can see, Donnan equilibrium is not applicable for animal cells, including neurons. However, it is theoretically possible to achieve equilibrium in conditions described above if an impermeant osmolyte were added to the external solution to create a so-called Double Donnan system [41]. Let us assume that the extracellular solution contains 135 mM of an impermeant neutral osmolyte, the same concentration as the internal impermeant anion (An^-^). To keep the same external osmolarity, [Na^+^]_o_ and [Cl^-^]_o_ have to be reduced from 150 mM to 82.5 mM. Once again, after opening the *gNa* and *gCl*, Na^+^, Cl^-^, and water will enter the cell and volume will increase. Again, [Na^+^]_i_ does not change, and [Cl^-^]_i_ increases when [An^-^]_i_ decreases, creating the illusion that An^-^ is being replaced by Cl^-^ (Fig 3A). And again, for equilibrium, both *E*_*Na*_ and *E*_*Cl*_ should be equal to *E*_*m*_. But in this case it is possible because [Na^+^]_o_ ≠ [Na^+^]_i_, and *E*_*Na*_ = -15.96 mV. After 30 min [Cl^-^]_i_ increases to 45.35 mM which corresponds to *E*_*Cl*_ = -15.98 mV, and the system is approaching equilibrium (Fig 3B). This is the Donnan equilibrium and when [Cl^-^]_i_ reaches 45.375 mM, the equation [Na^+^]_i_ * [Cl^-^]_i_ = [Na^+^]_o_ * [Cl^-^]_o_ will be true. Of course, entering Na^+^, Cl^-^, and water will increase the cell volume, but it stabilizes at 129% of the initial value.

Thus, in a Double Donnan system equilibrium can be achieved without compromising osmotic balance if an impermeant external neutral osmolyte is present in a sufficient concentration. The problem is that in order to keep [Cl^-^]_i_ low (and make osmotic room for important internal anions like proteins and nucleic acids) the concentration of the external neutral osmolyte must be high. However, in reality the total concentration of all external impermeant neutral osmolytes is quite low. Glucose is by far the most concentrated neutral osmolyte in ECS (5–6 mM). Numerous others have concentrations of small fractions of mM, and the total concentration of all external impermeant neutral osmolytes in normal conditions hardly ever exceeds 10 mOsm. But with only 10 mOsm of the neutral osmolyte, [Na^+^]_o_ and [Cl^-^]_o_ will be 145 mM, so according to the Donnan equation [Cl^-^]_i_ will be 140.17 mM, leaving almost no osmotic room (less than 10 mM) for other internal anions including vitally important proteins and nucleic acids.

The most concentrated substance outside of the cell is Na^+^. If the membrane were impermeant to Na^+^, the Double Donnan system could be created by utilizing Cl^-^ and another permeant cation, K^+^ (Fig 3C and 3D). Initial concentrations for these simulations are: [Na^+^]_o_ = [Na^+^]_i_ = 145 mM, [K^+^]_o_ = [K^+^]_i_ = 5 mM, [Cl^-^]_o_ = 150 mM, [Cl^-^]_i_ = 15 mM, and [An^-^]_i_ = 135 mM. When *gCl* and *gK* are open, both ions will enter the cell as in previous simulations. Water will follow and increase the cell volume and decrease the concentration of impermeant substances, which are [Na^+^]_i_ and [An^-^]_i_ in this case (Fig 3C). After approximately 5 minutes, the equilibrium potential for K^+^ decreases and the equilibrium potential for Cl^-^ increases to the same level as membrane potential, *E*_*K*_ = *E*_*Cl*_ = *E*_*m*_, (Fig 3D), and equilibrium is reached. The equilibrium potential for Na^+^ is different from the membrane potential, but it has no consequence since we assumed that the membrane is not permeable to Na^+^. Needless to say, this is purely theoretical and an absolutely unrealistic case, because every neuron has a significant Na^+^ conductance, and the presence of even the smallest Na^+^ conductance makes this equilibrium unachievable (see S2 Fig).

Concluding this part, it should be noted that the equilibrium conditions described above were determined completely by concentrations of the ions involved. The values of their conductances influence the time to achieve equilibrium but have no effects on the equilibrium potentials. Accordingly, when equilibrium is reached it cannot be changed by alterations of conductances.

### Cations, the pump, and cost of maintaining the membrane potential

The simulations in this section deal with two cations, Na^+^ and K^+^, and consequences of their *nonequilibrium* distribution across the cell membrane due to activity of the Na^+^/K^+^-ATPase. It has been recognized for more than a half century that “the big triad” of Na^+^ conductance, K^+^ conductance and Na^+^/K^+^-ATPase not only determines the membrane potential but forms the basis of the whole system of ionic homeostasis. Accordingly, the previous models concerning cell volume regulation starting from the early works [41–43] and up to the most recent [23–25] paid considerable attention to the cation conductances and Na^+^/K^+^-ATPase. Nevertheless, some features of the big triad were overlooked, and some others were misinterpreted.

The transmembrane redistribution of Na^+^ and K^+^ by the Na^+^/K^+^-ATPase is illustrated in Fig 4A. Before time = 0, [Na^+^]_o_ = [Na^+^]_i_ = 145 mM and [K^+^]_o_ = [K^+^]_i_ = 5 mM. Also 150 mM of an impermeant monovalent anion was present both inside and outside of the cell for electrical and osmotic balance. Since in these calculations the membrane is permeable to nothing but Na^+^ and K^+^, it does not matter what kind of anion it is (Cl^-^ or something else), but it is important that this anion is monovalent to preserve osmolarity-charge symmetry. In an attempt to make the cation transfer electrically neutral, an imaginary *electroneutral* Na^+^/K^+^-ATPase, which exchanges 3 Na^+^ for 3 K^+^ was used in this simulation. Also, *gNa* was equal to *gK*. At time = 0 the pump starts to transfer Na^+^ out of the cell and K^+^ into the cell against growing concentration gradients for both cations. Na^+^ starts to leak back to the cell and K^+^ - out of it, and with changes of the cation intracellular concentrations these leaks increase. At the same time the active transport of Na^+^ and K^+^ by the pump slows down because it strongly depends on [Na^+^]_i_ (see Methods), which is decreasing. The pump *rate* was set high (24 billion transfers/sec) and the pump *activity* with initially high [Na^+^]_i_ was 20.4 billion transfers/sec. (For explanation of our definitions of *the pump rate* and *the pump activity* see Methods). The pump activity decreased to 3 billion transfers/sec in about 1.5 seconds when [Na^+^]_i_ decreased to 8 mM and after about 5 seconds it stabilized at 331.4 million transfers/sec when [Na^+^]_i_ decreased to 2.52 mM. At this time the active transport of the pump and passive leaks of Na^+^ and K^+^ were equal, and steady state (when the concentrations, and consequently *E*_*m*_, remain the same) was achieved. It is important to emphasize that this is not an equilibrium state like Donnan equilibrium, because both *E*_*Na*_ and *E*_*K*_ are different from *E*_*m*_, and this stable state must be supported by constant energy expenditure.

Interestingly, in spite of the effort to make transport electrically neutral, *E*_*m*_ also changed, first decreasing to -27.35 mV and then increasing and stabilizing at +8.90 mV. The reason for *E*_*m*_ changes in a system with seemingly equal exchange of Na^+^ for K^+^ is the asymmetrical effect of the same absolute changes in [Na^+^]_i_ and [K^+^]_i_ on their respective equilibrium potentials. Indeed, when [K^+^]_i_ increases by 5 mM (from 5 to 10 mM), [K^+^]_i_ is doubled and *ΔE*_*K*_ = -18.5 mV; the simultaneous decrease of [Na^+^]_i_ by 5 mM (from 145 to 140 mM) means a relative change of only 3.4% and *ΔE*_*Na*_ < 1mV. The different time courses of *E*_*K*_ and *E*_*Na*_ (Fig 4A) illustrate this asymmetry, contrasting with symmetrical changes of [Na^+^]_i_ and [K^+^]_i_.

In the model, the activity of the pump is conveniently expressed in cycles per second and, accordingly, in ATP spent per second. So, we can directly connect the energy spent for the active transport of Na^+^ and K^+^ with the final intracellular concentrations of these ions in steady state and the resulting *resting E*_*m*_. The steady state values of concentrations and *E*_*m*_ that are reached after a few seconds in the simulation of Fig 4A were the result of a particular rate of steady state ATP utilization (331.4 million ATPs/sec). Additional simulations with larger or smaller initial pumping rates were done and yielded different sets of values of steady state concentrations and *E*_*m*_. This allowed the creation of the graph (Fig 4B) on which steady state values of [Na^+^]_i_, [K^+^]_i_ and *E*_*m*_ were plotted against an energy cost expressed in ATP spent per second. In subsequent figures lines through symbols will be used for graphs of this type; each set of points associated with a certain abscissa value represents a separate simulation. For instance, the concentrations and *Em* from simulations presented in Fig 4A are included in Fig 4B and marked by the arrows.

As more energy is spent, stronger electro-chemical gradients for Na^+^ and K^+^ are created. But as shown above, the stronger electro-chemical cation gradients do not necessary mean more negative *E*_*m*_. With the pump activity at 149.8 million ATP/sec, [K^+^]_i_ = [Na^+^]_i_ = 75 mM and *E*_*m*_ reached its most negative value (-27.35 mV) for this condition. With the pump activity at 299.7 million ATP/sec, the cation concentrations are exactly reversed from the extracellular values ([K^+^]_i_ = 145 mM and [Na^+^]_i_ = 5 mM); accordingly, *E*_*Na*_ = -*E*_*K*_, and *E*_*m*_ = 0 mV. We regard the expenditure of ATP on the rising side of the U-shaped *E*_*m*_ curve to be wasteful, because the same *E*_*m*_ could be achieved at a lower ATP cost at a point on the falling side of the *E*_*m*_ curve. Thus, we call the range of ATP utilization above the point of minimum *E*_*m*_ “overpumping.”

In reality, the Na^+^/K^+^-ATPase is of course electrogenic, transferring 3 Na^+^ for 2 K^+^, and that directly influences *E*_*m*_ (Fig 4C). Simulations with all the same conditions as previously, but with an electrogenic 3Na^+^/ 2K^+^ pump, show that *E*_*m*_ is equal not to 0, but to -17.98 mV at the point of the reversal of the cation concentrations. To reach this point, activity of the pump must be 359.6 million ATP/sec, i.e. the pump generates a current of 57.5 pA (3.596*10^8^ multiplied by the charge of one cation, which is 1.6*10^−19^ coulomb). Multiplication of this current by the input resistance of the modeled cell (312.5 MΩ) gives us the same voltage (-17.98 mV) that was calculated by the model using only the transmembrane movements of mass and charge. *E*_*Na*_ and *E*_*K*_ calculated from [Na^+^]_i_ and [K^+^]_i_ that were determined in simulations for Fig 4C, the voltage generated by the pump (*E*_*pump*_), and the final *E*_*m*_, are plotted against the pump activity in Fig 4D. To calculate *E*_*m*_ (under the modeled condition of equal *gNa* and *gK*) we used the equation:

$$E_{m}=(E_{Na}+E_{K})/2+E_{pump}$$

This analytically calculated *E*_*m*_ is equal to the *E*_*m*_ calculated by the model with precision better than 3*10^−5^ mV. Since the program calculates *Em* from the total intracellular electrical charge and the membrane capacitance (see Methods, Eq24) this fit provides another validation of the program.

Obviously, the cost of Na^+^ and K^+^ electro-chemical gradients and the resulting *Em* depends on the intensity of cation leakage. The cell with half the *gNa* and *gK* (input resistance = 625 MΩ instead of our usual 312.5 MΩ) will spend half the energy for the same gradients and voltage, and this is true for both an electroneutral and electrogenic pump (see S3 Fig).

The next step toward a more realistic system is considering the fact that the mean valence of impermeant anions is probably never equal to -1. The value of the mean valence of impermeant anions is difficult to determine experimentally; it probably varies in different cell types and possibly also in the same cells in different conditions. It also can be defined differently (more on this in Discussion). In this work we define the mean valence of the impermeant anions as the total charge of all impermeant intracellular anions divided by their total osmolarity. An exception will be made for Cl^-^, which is only temporarily, in this set of simulations, assumed to be impermeant; so, Cl^-^ is counted separately from the impermeant anions. For the simulations presented in Fig 4E we assumed that the mean valence = -3 to show what is probably the largest effect. When the mean valence of impermeant anions is larger than -1 (here and later, when we say “larger” in respect to mean valence of an anion we refer to the absolute value, ignoring the sign), fewer anion molecules are necessary to electrically compensate the cations. For instance, 150 mM of monovalent cations from the previous simulation can be neutralized by 45 mM of anions with valence = -3 in addition to 15 mM of Cl^-^. However, this would result in an intracellular osmolarity of only 210 mOsm/L. Then, to keep internal and external osmolarity equal, all intracellular concentrations should be proportionally increased by a ratio of 300/210, so the conditions for Fig 4E when the pump rate is zero are [Na^+^]_i_ = 207.14 mM, [K^+^]_i_ = 7.14 mM, [Cl^-^]_i_ = 21.43 mM and [An^-^]_i_ = 64.29 mM. Since intracellular concentrations of Na^+^ and K^+^ are higher than their respective extracellular concentrations, the membrane is hyperpolarized to -9.5 mV; this is an equilibrium state because *E*_*m*_ = *E*_*Na*_ = *E*_*K*_, and support of those unequal transmembrane distributions of Na^+^ and K^+^ as well as negativity of *E*_*m*_ does not cost any energy.

It is notable, comparing the results of calculations presented in Fig 4E (when the mean valence of the impermeant anions was = -3) with that in Fig 4C (when the mean valence of impermeant anions was = -1), that increasing the mean valence of impermeant anions shifts down the whole curve of *E*_*m*_ by the same value (-9.5 mV in this case) regardless of the pump activity. In both cases the most negative *E*_*m*_ was achieved at the pump activity of 201.5 million ATP per second. Also, in both cases the same amount of energy (359.6 million ATP per second) is necessary to reverse intracellular Na^+^ and K^+^ concentrations, although those reversed concentrations were different (see Fig 4C and 4E).

Fig 4F shows that similar effects were found in a condition when 15 mM of neutral impermeant osmolyte was added externally (the mean valence of intracellular impermeant anions was = -1 in this simulation). This increase of external osmolarity by 5% requires a proportional increase of all intracellular concentrations (including Na^+^ and K^+^), which in turn leads to generation of a small *E*_*Na*_ = *E*_*K*_ = *E*_*m*_ = -1.3 mV in the equilibrium state with no pump activity, as well as a downward shift of the *E*_*m*_ curve by -1.3 mV throughout the whole range of pump activity. Again, the same amount of energy as in previous simulations was needed to reach the critical point of the most negative *E*_*m*_ and reversed [Na^+^]_i_ and [K^+^]_i_. (see Fig 4C, 4E and 4F).

In all simulations of this part so far, Na^+^ and K^+^ have had the same conductance of 1*10^10^ ions/(sec*V), which is equal to 1.6 nanosiemens, resulting in a realistic input resistance of our modeled cell (312.5 MΩ). But it is typical for neurons that their *gK* is several times larger than *gNa*. For next set of simulations *gK* = 1.8*10^10^ and *gNa* = 2*10^9^ ions/(sec*V), so the input resistance remains the same, but the ratio *gK* : *gNa* is 9 : 1. The results of calculations performed with “simplified” conditions (mean valence of internal impermeant anions = -1, concentration of external impermeant osmolyte = 0) are presented in Fig 5A. They are clearly different from the results of calculations performed with the same conditions, but with *gK* = *gNa* (Fig 4C). First, as expected, *E*_*m*_ is significantly more negative. According to the chord conductance equation [44]

$$E_{m}=(gK*E_{K}+gNa*E_{Na})/(gK+gNa)$$

an increase of the K^+^ contribution makes *E*_*m*_ more negative. Interestingly, even in this case *E*_*m*_ can be still “overpumped” i.e. the negativity of *E*_*m*_ diminishes when the pump activity grows too large. Presumably there is little or no advantage in spending this energy for the pump when it leads to a smaller value of *E*_*m*_. Second, a high *gK* : *gNa* ratio enables the pump to spend less energy for creating ionic gradients. For instance, 179.8 million ATP per second is needed to equilibrate [Na^+^]_i_ and [K^+^]_i_ (both equal to 75 mM) when *gK* = *gNa* (Fig 4C), but only about 55 million ATP per second is sufficient when *gK* : *gNa* = 9:1 (Fig 5A).

Repeating these calculations with “realistic” conditions (mean valence of internal impermeant anions = -1.5, concentration of external electrically neutral impermeant osmolyte = 6 mM) gives results presented in Fig 5B. After necessary osmotic adjustments, [Na^+^]_i_ and [K^+^]_i_ in the equilibrium stage (pump activity = 0) increased to 174 and 6 mM, respectively, and the membrane hyperpolarized to *E*_*m*_ = -4.87 mV. Accordingly, compared to “simplified” conditions (Fig 5A), the range of cation concentration changes is wider and the whole *E*_*m*_ curve is shifted down by -4.87 mV. The terms “simplified” and “realistic” appear in quotation marks as a reminder that we use them only with respect to different concentration configurations that lead to symmetrical (convenient in calculations) or asymmetrical (usual in nature) osmolarity-charge configurations, respectively. Here both conditions are tested in a purely theoretical case where there is no Cl^-^ conductance, and later they will be applied to much more real situations with Cl^-^ conductance present and intracellular Cl^-^ concentration affected by cation-Cl^-^ cotransporters.

With many differences described above, all graphs of this part have one thing in common–the changes of [Na^+^]_i_ were always mirrored by the changes of [K^+^]_i_, i.e. all removed Na^+^ was replaced with an equal quantity of K^+^. This is true even if the only job of ATPase was to remove Na^+^ (3Na^+^ per ATP in this simulation) without transferring any K^+^. As soon as both [Na^+^]_i_ and *Em* decrease due to the electrogenic 3Na^+^-ATPase, Na^+^ begins to leak back to the cell and K^+^ also enters the cell attracted by the negativity. After some time a steady state will be established when K^+^ will be in equilibrium (*E*_*K*_ = *Em*) and the passive leak of Na^+^ into the cell will be equal to the active Na^+^ pumping. The larger the activity of the pump, the stronger the electro-chemical Na^+^ gradient, and the more Na^+^ is replaced with K^+^ in the cell, as illustrated by Fig 5C (“simplified” conditions) and 5D (“realistic” conditions). Here all energy was spent for the Na^+^ gradient; K^+^ was distributed passively. But still K^+^ plays key role - replacing Na^+^ it makes possible creating the Na^+^ gradient. If *gK* = 0, i.e. K^+^ cannot enter the cell, [Na^+^]_i_ would remain practically the same regardless of the activity of a pump moving only Na^+^. In this case all energy of the pump will be spent on generating a negative *Em*, which drives back all Na^+^ that was actively removed.

For better comparison of the effects that different parameters of the modelled cell have on the relation between Na^+^/K^+^-ATPase activity and *E*_*m*_, the results of several calculations are presented together in Fig 5E. A pair of simulation conditions—“simplified” and “realistic”–was used in each of four general settings. In Setting 1 an electrogenic 3Na^+^/2K^+^ pump was used and *gK* = *gNa* with the input resistance = 312.5 MΩ (as in Fig 4C). Other settings were different from Setting 1 in one of the following respects - imaginary electroneutral 3Na^+^/3K^+^ pump in Setting 2 (as in Fig 4B), ratio *gK* : *gNa* = 9:1 in Setting 3 (as in Fig 5A and 5B), and imaginary only Na^+^ pump (3Na^+^ per ATP) in Setting 4 (as in Fig 5C and 5D). Thus, it is proper to compare Setting 1 with the three others. As expected, an electroneutral 3Na^+^/3K^+^ pump (Setting 2) had a smaller effect on *E*_*m*_ than an electrogenic 3Na^+^/2K^+^ pump (Setting 1); what was interesting is that the difference was not large before the pump activity reached the level of overpumping. From the energy point of view the most interesting result comes from comparing Setting 1 with Setting 3 - high *gK* relative to *gNa* enabled the pump to create stronger ionic gradients and promote a more negative *E*_*m*_ while spending less ATP. A strongly electrogenic 3Na^+^ pump (Setting 4) significantly hyperpolarized the membrane at high pump activities, but it came with a high ATP price if *gK* = *gNa*. It is also notable that under every setting and with every pump activity the difference between the “simplified” and “realistic” conditions was always the same and equal to what was it was in the passive, no pump condition, i.e.-4.87 mV.

As we already pointed out, in all simulations in this section the internal Na^+^ was replaced with an almost exactly equal quantity of the external K^+^ regardless of relative conductance of these ions and the stoichiometry of the pump. Here we would like to emphasize the word “almost,” since the exchange of Na^+^ for K^+^ was not exact. The negative *E*_*m*_ means that there is some deficiency of internal cations. Similarly, when ions were overpumped sufficiently for *E*_*m*_ to become positive, it is due to some surplus of internal cations. Subtraction or addition of an intracellular substance leads to osmotic imbalance, compensatory water movement, and consequently, appropriate cell volume changes. The volume changes associated with *E*_*m*_ changes from Fig 5E are presented in Fig 5F. As expected, dependence of the cell volume on the pump activity closely followed the dependence of *E*_*m*_ on the pump activity in every setting. Since all volumes were normalized to the initial cell volume in the passive state with no pumping (defined as 100%), the pre-existing cation deficiency associated with initial negativity of *E*_*m*_ in the “realistic” conditions (i.e. the -4.87 mV) was already counted; so, the curves for the volume changes in the “simplified” and “realistic” conditions are identical in all four settings.

The main result related to the cell volume, however, is that its changes are extremely, unnoticeably small. A tiny quantity of ions is needed to recharge the cell membrane significantly. A simple calculation shows that 9.6*10^−13^ coulomb of charge will hyperpolarize our modelled cell, with membrane capacitance 1.2*10^−11^ farad, to -80 mV. This charge is carried by 6*10^6^ ions, which equals 13.3 μM in our cell with volume 7.5*10^−13^ L, i.e. only 0.0044% of the total internal osmolarity. The results of simulations presented in Fig 5C and 5D demonstrate precisely that: when the cell was hyperpolarized to about -80 mV (setting 3), the cell volume change was a bit more than 0.004%. Thus, we can conclude that the Na^+^/K^+^ pump replaces internal Na^+^ with practically the same quantity of K^+^. Accordingly, the pump has practically no measurable effect on the cell volume, and this is true with any stoichiometry of the pump and conductance of Na^+^ and K^+^.

To summarize this part, we can conclude that in the “only cation” system tested above three properties - Na^+^ conductance, K^+^ conductance and Na^+^/K^+^-pump activity - determine with certainty the values of three features - [Na^+^]_i_, [K^+^]_i_ and *E*_*m*_. When the properties are constant, the determined features also stay unchanged in steady or resting state. But it is not an equilibrium state, and a constant expenditure of energy is required to keep it. Changes in the pump activity lead to changes in [Na^+^]_i_, [K^+^]_i_ and *E*_*m*_ until a new steady state is reached. The same is true for changes of Na^+^ or K^+^ conductances. It is also important to note that changes in the pump activity as well as changes in cation conductances have no practical effects on the cell volume.

### Conductance of Cl^-^ and cell volume

In this part, the cation system described above will be enriched by addition of Cl^-^ conductance and later by Cl^-^-cation cotransporters. Cl^-^ is by far the most concentrated external anion and numerous Cl^-^ permeable channels and Cl^-^ transferring transporters make this ion unavoidably important for the nervous system. Here we will show that Cl^-^ conductance is *the* reason for voltage-related cell volume changes.

The results presented in Fig 6 illustrate the changes in concentrations, voltage and cell volume when Cl^-^ conductance was opened at time = 0 to disturb the resting state achieved with open cation channels and active Na^+^/K^+^-ATPase (*gNa* = 8*10^9^ ions/(sec*V), *gK* = 1.2*10^10^ ions/(sec*V), pump activity 2.64*10^8^ ATP/sec, *E*_*m*_ = -43.3 mV). This resting state, with a relatively high *gNa* and a moderate *Em*, was chosen so that changes in *gCl* could potentially cause either depolarization or hyperpolarization. As a result of this disturbance [Cl^-^]_i_ changed significantly, but [Na^+^]_i_, and [K^+^]_i_, changes were barely noticeable (Fig 6A) and only transient (Fig 6B). Regardless of the initial [Cl^-^]_i_, (15 mM for solid and dashed lines, and 45 mM for the dotted line) or the value of *gCl* (10^11^ ions/(sec*V) for the solid line and 10^10^ ions/(sec*V) for the dashed and dotted lines), [Cl^-^]_i_ in the new resting state was 29.6 mM. At this concentration *E*_*Cl*_ = -43.3 mV, i.e. *E*_*Cl*_ is equal to the *E*_*m*_ established by the cations. To electrically compensate transmembrane movement of Cl^-^, Na^+^ and K^+^ move together with it, entering the cell when [Cl^-^]_i_ increases from 15 to 29.6 mM and leaving the cell when [Cl^-^]_i_ decreases from 45 to 29.6 mM. These ionic movements cause the cell volume to increase in the former case and decrease in the latter case (Fig 6A) keeping cation concentrations constant. The intracellular concentration of impermeant anion An^-^ experiences opposite changes to Cl^-^ as a result of the volume change, keeping [Cl^-^]_i_ + [An^-^]_i_ almost constant. The small deviations of total anion concentration (~0.25 mM and ~0.55 mM with *gCl* of 10^10^ ions/(sec*V) and 10^11^ ions/(sec*V), respectively) from the initial value of 150 mM that peaked in the first 2–3 seconds after increasing *gCl* was a result of restricted transmembrane water movements assumed in this modeling. There was no such deviation if we assumed instant water transfer.

Some small changes of [Na^+^]_i_ and [K^+^]_i_, as well as of *E*_*m*_, also happened after opening of Cl^-^ conductance, but, in contrast to anion concentrations and volume, both cation concentrations and *E*_*m*_ recovered to their original values in 5–6 minutes when the system reached the new steady state (Fig 6B). In the case of low initial [Cl^-^]_i_ (15 mM, dashed lines) *E*_*m*_ was temporary hyperpolarized, Cl^-^ and both cations entered the cell, and the cell volume increased. Initially Na^+^ entered the cell faster than the volume increase and [Na^+^]_i_ slightly increased; at the same time [K^+^]_i_ decreased (in spite of the influx of K^+^) and the sum of [Na^+^]_i_ and [K^+^]_i_ remained practically equal to 150 mM, with small deviations in the first seconds due to limited water transfer, as in the case of the anions. The transient increase of [Na^+^]_i_ and the decrease of [K^+^]_i_ are due to the stronger diluting effect of increasing volume on the ion in higher concentration, which is K^+^. Opposite transient changes in *E*_*m*_, [Na^+^]_i_ and [K^+^]_i_ occurred if [Cl^-^]_i_ started at 45 mM (Fig 6B, dotted lines). If the steady state condition before opening *gCl* had been set to achieve [Na^+^]_i_ = [K^+^]_i_ = 75 mM (at a pump activity 1.66*10^8^ ATP/sec), opening of *gCl* would have led to a decrease of [Na^+^]_i_ and an increase of [K^+^]_i_, because K^+^ conductance in these simulations was set to be larger than Na^+^ conductance. If the initial steady state condition had been [Na^+^]_i_ = [K^+^]_i_ = 75 mM and *gNa* = *gK* (at the pump activity 1.80*10^8^ ATP/sec), opening of *gCl* would have had no effect on the cation concentrations, besides small and short-lived deviations (both increases) related to delayed water movements, and it would have been the same for electrogenic and neutral pumps. *gCl* affected nothing except the time necessary for equilibration; it took 6–7 minutes with *gCl* = 10^10^ ions/(sec*V) (dashed and dotted lines in Fig 6) and 3–4 minutes with *gCl* = 10^11^ ions/(sec*V) (solid lines in Fig 6).

Calculations presented in Fig 6, parts A and B were done in “simplified” conditions, i.e. assuming that the mean valence of internal impermeant anions = -1 and that there were no external osmolytes besides Na^+^, K^+^ and Cl^-^. Largely similar results were obtained in “realistic” conditions (the mean valence of internal impermeant anions = -1.5 and the concentration of external electrically neutral impermeant osmolytes = 6 mM) with the most notable difference being in the cation concentrations (Fig 6C and 6D). Smaller amounts of polyvalent intracellular anions were needed to electrically compensate intracellular cations and that, together with the addition of impermeant extracellular osmolyte, demanded certain osmotic adjustments that affected the initial ionic concentrations. As a result, the resting [Na^+^]_i_ and [K^+^]_i_ were different from those in “simplified” conditions described above with the same rate of the Na^+^/K^+^-ATPase. Moreover, [Na^+^]_i_ and especially [K^+^]_i_ were different depending on how much of the initial anion concentration was due to [Cl^-^]_i_ (10% and 30% of the total charge of intracellular anions for the dashed and dotted lines, respectively). Accordingly, *E*_*m*_ also was different under low or high initial [Cl^-^]_i_, although the difference was only ~1 mV (Fig 6D). In this “realistic” condition, just as in the previously described “simplified” condition, opening of *gCl* led to changes of [Cl^-^]_i_ toward a new value that was the same regardless of initial [Cl^-^]_i_. Again, the transmembrane movement of Cl^-^ was accompanied by co-directed movements of Na^+^ and K^+^ that led to appropriate changes in the cell volume and consequently [An^-^]_i_ (Fig 6C). However, the absolute change of [An^-^]_i_ was 1.5 times smaller than the change of [Cl^-^]_i,_ since each An^-^ was carrying 1.5 times more charge. In this condition, [Na^+^]_i_ and [K^+^]_i_, and accordingly *E*_*m*_, were also shifted to new levels (Fig 6D). When a new resting state was established, *E*_*m*_ and all four cation and anion concentrations stabilized at new values which were not dependent on initial [Cl^-^]_i_. And the new [Cl^-^]_i_ was again exactly what was required to make *E*_*Cl*_ = *E*_*m*_ (-46.15 mV).

Since Cl^-^ is in equilibrium in this new resting state, alterations in *gCl* cannot change anything in the system. But alterations in conductances of nonequilibrated cations can, and some of the results are different depending on *gCl*. Fig 7 illustrates how temporal changes of *gNa* affect the ionic concentrations, cell volume and voltage under various *gCl*. The cell modelled here is permeable to Na^+^, K^+^, and Cl^-^ and is at rest under “realistic” conditions. So, it is similar to the one presented in Fig 6C and 6D, but with one difference in Fig 7A and 7B: it uses the imaginary electroneutral 3Na^+^/3K^+^-ATPase that does not transfer any net charge or mass and consequentially cannot directly influence the cell volume or voltage.

At time = 0 *gNa* was reduced by a factor of 4 (from 8*10^9^ to 2*10^9^ ions/(sec*V)) and 10 seconds later *gNa* returned to its original value. The temporary decrease of *gNa* leads to a hyperpolarization of *E*_*m*_, moving it closer to *E*_*K*_. This reduces the driving force for K^+^, and passive K^+^ efflux decreases. Passive Na^+^ influx also decreases (in spite of the increased driving force for Na^+^) due to the reduction in *gNa*. But the Na^+^/K^+^-ATPase continues to pump K^+^ in and Na^+^ out of the cell initially with the same activity. As a result, [K^+^]_i_ increases and [Na^+^]_i_ decreases during the temporary decrease of *gNa*. These cation changes are almost identical when *gCl* is negligible (10^8^ ions/(sec*V), i.e. less than 0.5% of the total transmembrane conductance, dashed lines) or considerable (10^10^ ions/(sec*V), i.e. more than 30% of the total transmembrane conductance, solid lines). *E*_*m*_ is more sensitive to *gCl* (Fig 7B), demonstrating the well-known “shunting inhibition” (see S2 Text), supported in many neurons by the Cl^-^ permeable GABA- and glycine-gated channels. But it is the cell volume that is affected most by the value of Cl^-^ conductance. The hyperpolarization evokes efflux of Cl^-^, which was in equilibrium before the time = 0. In order to electrically compensate it, an efflux of cations is required. The model shows that under these conditions Na^+^ influx decreases more than K^+^ efflux, causing a net efflux of cations that is equal to Cl^-^ efflux, and when the ions leave the cell, the cell volume decreases. Depending on the value of *gCl*, the ionic fluxes could be large or small, determining the size of volume changes. Thus, during electrical activity associated with changes of cation concentrations the cell may or may not experience detectable changes of its volume, depending on the value of *gCl*.

It should be noted that the decrease of [Na^+^]_i_ slows down the Na^+^/K^+^-ATPase. The simulations in Fig 7A and 7B used an electroneutral 3Na^+^/3K^+^-ATPase, and alterations in the pump activity had no consequences for the cell volume and voltage. But the real electrogenic 3Na^+^/2K^+^-ATPase does transfer both charge and mass, and it is intuitively expected that the electrogenic pump should contribute to changes of *E*_*m*_ and the cell volume. The results of modeling with an electrogenic Na^+^/K^+^ pump are presented in Fig 7C and 7D. As expected, decrease of the pump activity associated with the decrease in [Na^+^]_i_ clearly manifested itself in a slow reduction of the hyperpolarization (Fig 7D). However, the volume changes were again completely under control of the *gCl*. Independently of the stoichiometry of the pump, changes in its activity cannot change the cell volume if the membrane is not permeable to Cl^-^, and on the other hand, when Cl^-^ conductance is considerable the volume changes happen irrespective of whether the pump is electroneutral or electrogenic.

One more point should be made concerning the relation between the Na^+^/K^+^-ATPase and the cell volume. Activity of the pump, and consequently the expenditure of ATP, follows [Na^+^]_i_. Since the changes of [Na^+^]_i_ were practically identical with high and low *gCl* (Fig 7A and 7C), the expenditure of ATP was the same and irrelevant to the volume changes. This also can be seen in the simulations of Fig 6. Changes of [Na^+^]_i_ tell us that some extra energy was spent during the transition when the cell volume increased (Fig 6 dashed lines) and some energy was saved when the cell volume decreased (Fig 6 dotted lines), but after reaching the resting state, the cell spent exactly the same amount of energy to keep the larger volume as to keep the smaller volume. And it also was equal to the amount of energy the cell spent before *gCl* opening, precisely in “simplified” conditions and with precision of a fraction of 1% in “realistic” conditions.

Certain molecular mechanisms can influence [Cl^-^]_i_, shifting it away from equilibrium, so that *E*_*Cl*_ ≠ *E*_*m*_. The most important of these for the nervous system are two cation-Cl^-^ cotransporters - the Na^+^,K^+^,2Cl^-^ cotransporter and the K^+^,Cl^-^ cotransporter (NKCC and KCC, respectively). Fig 8A shows steady state values of intracellular ion concentrations, *E*_*m*_, and cell volume as a function of the rate of the NKCC, in the condition when Cl^-^ conductance is very small (10^8^ ions/(sec*V)). The lower row of numbers under the x-axis represents the corresponding *activity* of NKCC, which gives information on the actual quantity of ions transferred across the membrane. As for the Na^+^/K^+^-ATPase, the activity, in distinction to the rate, is dependent on ionic concentrations and will change together with them. The activity of the transporter is *k*_*1*_ times its rate (see Methods), where

$$k_{1}=log10\;\left(\left({\left[Na^{+}\right]}_{o}*{\left[K^{+}\right]}_{o}*{\left[Cl^{‑}\right]}_{o}{}^{2}\right)/\left({\left[Na^{+}\right]}_{i}*{\left[K^{+}\right]}_{i}*{\left[Cl^{‑}\right]}_{i}{}^{2}\right)\right).$$ (35)

The NKCC pumps Na^+^, K^+^, and Cl^-^ into the cell, and the direct result of that is an increase in cell volume. The higher the cotransporter rate (and activity), the larger the cell volume (Fig 8A). [Cl^-^]_i_ also increases with the cotransporter rate, but, interestingly, [Na^+^]_i_ and [K^+^]_i_ remain almost exactly the same (note: there is a 3:2 Na^+^/K^+^-ATPase in these simulations). [Na^+^]_i_ increased by only 0.006 mM and [K^+^]_i_ actually decreased by 0.005 mM. *E*_*m*_ also changed very little–from -43.3 mV to -43.2 mV, reflecting a subtle depolarizing influence of Cl^-^, which is not in equilibrium in this case. At the highest activity of the cotransporter, [Cl^-^]_i_ reached 82.98 mM; at this concentration *E*_*Cl*_ = -15.8 mV. But the conductance of Cl^-^ in this simulation was very low, so the Cl^-^ contribution to *E*_*m*_ is negligible.

Fig 8A also clearly demonstrates that the capability of NKCC to elevate [Cl^-^]_i_ (and the cell volume) is limited. When [Cl^-^]_i_ is increasing, *k*_*l*_ approaches 0. Because the cation concentrations are constant (Fig 8A) and so is [Cl^-^]_o_, this limiting value of [Cl^-^]_i_ can be obtained by setting *k*_*l*_ = 0 and solving the Eq 35 for [Cl^-^]_i_. In our calculations [Na^+^]_o_ = 145 mM, [K^+^]_o_ = 5 mM, [Na^+^]_i_ = 17.9 mM, [K^+^]_i_ = 132.1 mM, and [Cl^-^]_o_ = 150 mM, so the largest [Cl^-^]_i_ that can be achieved is 83.06 mM. [Cl^-^]_i_ approaches this level when the cotransporter rate is 10^8^ cycles/sec and activity = 1.35 million cycles/sec. At that point the driving force of the transporter is almost exhausted, and increasing its rate by 100 times means increasing activity only to 1.37 million cycles/sec, i.e. only by 1.5%.

Fig 8B shows how the activity of NKCC influences concentrations, voltage and volume when Cl^-^ conductance is high (10^10^ ions/(sec*V)). In such conditions the cotransporter is similarly capable of elevating [Cl^-^]_i_ and increasing the cell volume to about the same values as in the case of low *gCl*, but the activity of the cotransporter has to be roughly 100 times higher because it has to overcome a leakage of Cl^-^ that is 100 times larger through the high conductance. More importantly, the high *gCl* makes Cl^-^ a noticeable contributor to *E*_*m*_. Thus, the cotransporter-generated increase of [Cl^-^]_i_ is accompanied not only by an increase in cell volume, but also by a depolarization from -43.3 mV to -34.6 mV. [Na^+^]_i_ and [K^+^]_i_ were again almost unaffected, although their small changes were larger than in the case of low *gCl*: +0.42 mM for [Na^+^]_i_ and -0.42 mM for [K^+^]_i_.

The KCC uses the strong K^+^ outward concentration gradient to extract Cl^-^ from the cell. The coefficient *k*_*2*_ that links the activity of the cotransporter to its rate is expressed as follow:

$$k_{2}=log\;\left(\left({\left[K^{+}\right]}_{o}*{\left[Cl^{‑}\right]}_{o}\right)/\left({\left[K^{+}\right]}_{i}*{\left[Cl^{‑}\right]}_{i}\right)\right).$$ (36)

According to this equation, when *k*_*2*_ = 0 the lowest [Cl^-^]_i_ which can possibly be achieved in our conditions ([K^+^]_o_ = 5 mM, [Cl^-^]_o_ = 150 mM, [K^+^]_i_ = 132.1 mM) is 5.68 mM, and the calculations show that our modeled cell approaches this limit with a cotransporter activity of 4.13 million cycles/sec when *gCl* is low (Fig 8C). Together with lowering of [Cl^-^]_i_, KCC decreased the cell volume, but the cation concentrations remained remarkably similar (only +0.040 mM for [Na^+^]_i_ and -0.037 mM for [K^+^]_i_), in spite of the fact that the cotransporter removed exactly the same amount of K^+^ as Cl^-^. *E*_*m*_ also was very little affected - the cotransporter at its maximal activity produced only -0.27 mV of additional hyperpolarization.

As expected, increasing *gCl* 100 times demanded much higher activity of the cotransporter for lowering of [Cl^-^]_i_ toward the limit (Fig 8D). Also, as expected for a high *gCl*, the decrease of [Cl^-^]_i_ caused by KCC was accompanied by significant hyperpolarization (from -43.3 mV to -59.7 mV). The cation concentrations were affected as well, although not as much as [Cl^-^]_i_: [K^+^]_i_ decreased by 2.6 mM and [Na^+^]_i_ increased by the same 2.6 mM.

It is also noteworthy that activity of both cation-Cl^-^ cotransporters is associated with increased expenditure of energy. Moving one more element of the system (Cl^-^) out of the equilibrium state obviously should cost some extra energy, regardless of the direction of this movement - an increase or decrease of [Cl^-^]_i_ and, consequently, an increase or decrease of the cell volume and depolarization or hyperpolarization of the cell membrane. In this respect, it is surprising how little extra energy was needed in the case of the NKCC. When [Cl^-^]_i_ increased by 177% and cell volume increased by 77% with a very active cotransporter, ATP consumption increased only by 2.3%. And this was under the high *gCl* condition. When *gCl* was low, even larger increases of [Cl^-^]_i_ and the cell volume were achieved with a tiny cost of 86,000 extra ATPs per second, which is 0.03% of the total energy. The KCC is more demanding. A decrease of [Cl^-^]_i_ to 19.7% of its initial concentration with a cell volume reduction to 83.5% required an additional 12% of ATP when *gCl* was high, and an extra 2% of ATP did comparable work when *gCl* was low.

All simulations of cation-Cl^-^ cotransporters above were done in the “simplified” condition. We performed the same series of calculations in the “realistic” condition and high gCl for NKCC (Fig 8E) and for KCC (Fig 8F). Changes of [Cl^-^]_i_ in an asymmetric concentration-charge system, like the “realistic” condition, is associated with some additional complications in cation concentrations. The [Na^+^]_i_ + [K^+^]_i_ is not constant anymore; contrarily, the sum must change due to changes in the ratio “total anion charge”/”total anion concentration” resulting from opposite changes in the concentrations of monovalent Cl^-^ and polyvalent An^-^ (more on this in Discussion). As a result, the logic of cation concentration behavior induced by cation-Cl^-^ cotransporters is not obvious. Specifically, NKCC noticeably decreased [K^+^]_i_, but did not change [Na^+^]_i_, in spite of pumping both cations into the cell. In its turn, KCC increased [Na^+^]_i_, although it did not transfer this ion; the cotransporter also had a biphasic increase-decrease effect on [K^+^]_i_. The movement of water and changes in *E*_*m*_ are important in understanding these unintuitive changes.

Effects of cation-Cl^-^ cotransporters on [Na^+^]_i_ and [K^+^]_i_ are intriguing and deserve a more detailed analysis in the future, but for the purpose of this paper it should be stressed that those effects were small. In most cases there were almost no cation changes compared with changes of Cl^-^, which was transferred simultaneously with cations and in equal amount. And [Cl^-^]_i_ changes were always accompanied by changes of the same sign in the cell volume. Low *gCl* allowed to the system to achieve large effects on [Cl^-^]_i_ and the cell volume at a small activity of the cotransporters, but high *gCl* was needed to influence *E*_*m*_. It seems that those cotransporter-evoked Cl^-^-dependent alterations of *E*_*m*_ are responsible for the disturbances in cation concentrations.

### Alterations of osmolarity and their consequences for ionic concentrations, voltage and volume

In the last part of this work we will examine how changes of external and internal osmolarity affect the cell volume, [Na^+^]_i_, [K^+^]_i_, [Cl^-^]_i_, [An^-^]_i_, and *E*_*m*_. The reason-consequence chain in this section will be different from the previous sections. Up to this point changes in concentration of permeant ions evoked changes in *Em* and possibly in cell volume, if the redistribution of ions was not osmotically balanced. Here, the initial event was an alteration of osmolarity that directly and predictably influences the cell volume. When *external* osmolarity increases, the cell shrinks; when *internal* osmolarity increases, the cell swells. These changes of cell volume may or (surprise!) may not lead to changes in intracellular ionic concentrations, as will be shown. Finally, changes of ion concentration, if they occur, will inevitably change *E*_*m*_.

Simulations in this part resemble what happens or may happen during a regulatory volume increase (RVI). The initial set of calculations simulates the first phase of RVI in which extracellular osmolarity is increased by adding some impermeant neutral osmolyte. The set includes simulations with high *gCl* (10^10^ ions/(sec*V), solid lines in Fig 9) and with low *gCl* (10^8^ ions/(sec*V), dashed lines in Fig 9), both in “realistic” conditions. The first phase of RVI lasts a few seconds to minutes [45], so 30 mM of an external osmolyte was added at the rate of 0.5 mM/sec for 1 minute (gray area in Fig 9A and 9B). Qualitatively all changes of the ion concentrations, the volume and the voltage were in accord with expectations. The cell shrank, and [Na^+^]_i_, [K^+^]_i_, [Cl^-^]_i_, and [An^-^]_i_ increased in proportion to their initial level, at least at first glance. *E*_*m*_ hyperpolarized, which was anticipated because the increase of [K^+^]_i_ enhanced the K^+^ transmembrane gradient and its hyperpolarizing effect, and the increase of [Na^+^]_i_ diminished the Na^+^ transmembrane gradient and its depolarizing effect. But the quantitative picture is more complicated. First, the cation increases were not proportionally equal. At the end of the first minute, when external osmolarity reached its maximum (336 mOsm. i.e. 9.8% more than the initial value of 306 mOsm), [K^+^]_i_ increased by more than 10% and [Na^+^]_i_ increased by a little more than 2%. This is an apparent deviation from the simplistic volume-induced increases of concentrations that were expected to be proportionally equal, and it points toward a redistribution of ions during osmosis-related changes.

Redistributions of Cl^-^ are the most interesting because Cl^-^ is tightly connected with cell volume. There were no cation-Cl^-^-cotransporters in this simulation, so Cl^-^ was in equilibrium before the increase of external osmolarity. Osmosis-related shrinkage of the cell increased [Cl^-^]_i_ and diminished the concentration-dependent inward-directed component of the driving force for Cl^-^. At the same time a cation-induced hyperpolarization enhanced the voltage-dependent outward-directed component of the driving force for Cl^-^. Thus, Cl^-^ left the cell, and the increase of [Cl^-^]_i_ was smaller than expected from the volume decrease itself. The difference, of course, depended on the value of *gCl*. When *gCl* was low, the increase of [Cl^-^]_i_ was close to the expected change from volume alone (9.5% vs 9.7%), but when Cl^-^ conductance was high, those two numbers were very different (3.1% vs 11%). After 1 minute of increased external osmolarity [Cl]_i_ was noticeably out of equilibrium. In the case of low *gCl*, [Cl^-^]_i_ after 1 minute was 28.10 mM; accordingly, *E*_*Cl*_ = -44.7 mV, i.e. 4.6 mV more positive than *E*_*m*_ (-49.3 mV). High *gCl* shunted the membrane, so the hyperpolarization was smaller (to -48.3 mV). The rise of [Cl^-^]_i_ also was smaller due to Cl^-^ leakage (to 26.47 mM), so *E*_*Cl*_ (-46.3 mV) was 2 mV more positive than *E*_*m*_. When external osmolarity stabilized, [Cl^-^]_i_ began to decrease, leading to both further hyperpolarization and a further volume decrease. If Cl^-^ conductance was high, Cl^-^ quickly equilibrated and after 10 minutes [Cl^-^]_i_ = 23.70 mM, i.e. about 2 mM less than the initial concentration. That new [Cl^-^]_i_ was at equilibrium and fit with new electrical conditions (*E*_*Cl*_ = *E*_*m*_ = -49.3 mV). If *gCl* was low, no changes in [Cl^-^]_i_, volume or voltage were visible from 1 to 10 minutes, except a small and quick depolarization that reflected cation adjustment after the disturbance. However, the calculations showed that after several hours the cell would come to the same equilibrium state as in the case of high *gCl*.

Knowing that the ions were redistributed during the osmotic shock, it is not surprising that the cell volume changes were themselves different from expectations. An increase of external osmolarity by 8.9% should decrease volume in a cell that is impermeant to anything but water by 8.2%, and that was close to the volume reduction at the end of osmotic shock when *gCl* was low (8.8%). But when Cl^-^ conductance was high, the cell volume decreased by 9.8% at 1 minute and by 11.8% in the eventual resting state.

Fig 9C and 9D demonstrates that fundamentally similar changes happen when NaCl, i.e. a substance that easily can cross the cell membrane, was used instead of a neutral impermeant osmolyte. As in the previous case, the external osmolarity was slowly elevated by 30 mOsm by the end of 1 minute; to do this NaCl was added at a rate of 0.25 mM/sec. Addition of extracellular NaCl directly influenced not only external osmolarity, but also the transmembrane gradients of Na^+^ and Cl^-^. Increasing the Na^+^ gradient enhanced the depolarizing effect of this cation on *E*_*m*_, which resulted in a smaller osmotic-dependent hyperpolarization (if compared to the case of an increased external neutral osmolyte described above), but only when *gCl* was low (compare dashed lines in Fig 9B and 9D). When *gCl* was high the hyperpolarization during the osmotic shock was even slightly larger. The increased transmembrane Cl^-^ gradient had more recognizable effects. Now Cl^-^ did not move very far from equilibrium, as in the case of a neutral osmolyte. The difference between *E*_*Cl*_ and *E*_*m*_ never exceed 0.8 mV with high *gCl*, and 1.5 mV with low *gCl*. As a result, [Cl^-^]_i_ experienced much smaller changes after the osmotic disturbance on its way to equilibrium. Accordingly, smaller further hyperpolarization and volume decreases happened after adding NaCl than a neutral osmolyte (Fig 9B and 9D). Again, a long time (many hours) is needed to reach equilibrium if *gCl* is low, which leads to the illusion that nothing changed in this case after the disturbance.

It also should be noted that some extra energy is needed to support steady state in a smaller cell volume after the external osmotic increase, although the cost is not high. ATP expenditure increased by about 1.5% when the system stabilized after the neutral osmolyte-induced disturbance, and about 3% extra ATP was needed in case of NaCl.

In the next two sets of simulations the internal osmolarity was increased. This was similar to the second, active phase of RVI. First, a neutral impermeant osmolyte was added to intracellular space (Fig 10A and 10B). Such an increase could happen when some macromolecules were broken down to many smaller molecules (like glycogen to glucose) or some osmolyte (for instance, taurine) was transported into the cell from the extracellular space with an appropriate transporter. For the simulation, we assume that a neutral impermeant osmolyte increases with a rate of 0.05 mM/sec for 10 minutes until its concentration reaches 30 mM. This might be too fast to be real, but slowing down the process does not change the results (except diminishing the difference between simulations with different *gCl*).

Most (but not all) changes of concentrations, voltage and volume associated with elevation of internal osmolarity are just opposite to those observed with a simulated increase of external osmolarity (Fig 10A and 10B). The cell swelled, and intracellular concentrations decreased, with the important exception of [Cl^-^]_i_, which increased when *gCl* was high. Again, decreases of ionic concentrations were not proportionally equal, in spite of the proportionality that would be expected as the direct effect of the increasing volume. [K^+^]_i_ decreased by more than 10%, while [Na^+^]_i_ decreased by less than 2%. *E*_*m*_ depolarized due to decreases in both cation concentrations. The depolarization forced Cl^-^ to enter the cell, but it had little effect, and [Cl^-^]_i_ still decreased when *gCl* was low. However, when *gCl* was high, this depolarization-driven Cl^-^ influx was substantial and [Cl^-^]_i_ increased. Accordingly, the difference between *E*_*Cl*_ and *E*_*m*_ was small (maximum 0.44 mV), and likewise the effect of Cl^-^ on *E*_*m*_ was also small. Still, Cl^-^ was out of equilibrium, and when the buildup was complete, [Cl^-^]_i_ continued to increase to equilibrate with the new *E*_*m*_, initiating a further depolarization and swelling. The new resting state was reached much more slowly with low Cl^-^ conductance. Finally, although it might cost energy to build up an intracellular osmolyte, the cell actually saved about 1.5% of the ATP required to support ionic balance with the new larger volume.

The last set of simulations dealt with the curious case of the buildup of an intracellular impermeant anion with a mean valence equal to -1.5. It should be noted that synthesis of *a new* organic anion must be accompanied by a cation for electroneutrality. The most probable cation in such a case is H^+^, so the addition of an anion would also cause the addition of an acid. The regulation of intracellular pH is an undoubtedly important, but complicated, problem that goes beyond the scope of this paper. So, we assume that our modeled cell is capable of resolving the problem of stabilization of pH. For instance, the cell could exchange each new internal H^+^ for external Na^+^ using a Na^+^/H^+^ exchanger. Thus, in our simulations the buildup of impermeant anion will be supplemented by appropriate addition of Na^+^. In the “realistic” conditions of our simulation 3 Na^+^ were needed to electrically compensate 2 An^-^, which have a mean valence = -1.5. Accordingly, buildup of the anion with rate of 0.02 mM/sec for 10 minutes was accompanied by addition of Na^+^ at 0.03 mM/sec, to produce osmotically the same increase as the electrically neutral osmolyte in the previous set of simulations.

The results of this buildup were a bit surprising (Fig 10C and 10D). Besides the inevitable increase of the cell volume, there were no other changes of significance. Addition of 12 mM of An^-^ with 18 mM of Na^+^ was largely compensated by the cell swelling, so [An^-^]_i_ and [Na^+^]_i_ increased only by 0.54 mM (0.5% of initial) and by 0.3 mM (1.6% of initial), respectively. [K^+^]_i_ decreased as expected, but only by 0.13 mM (less than 0.1% of initial). [Cl^-^]_i_ experienced the largest relative changes (2.5%), which still was less than 1 mM. The small changes in ion concentrations produced small changes in *E*_*m*_ (-0.46 mV). And most importantly, all concentrations, including [An^-^]_i_ and [Na^+^]_i,_ returned to their initial values in a few minutes after the end of the buildup. Together with ions, *E*_*m*_ also returned to its initial value. Thus, the lone result of addition of AnNa_x_ was increase of the cell volume. Some extra energy was spent during swelling, but when ionic gradients were restored, exactly the same amount of ATP could support the resting state at a larger cell volume.

## Discussion

The model presented here is very flexible, and allows calculation of both dynamic and steady state values for cell volumes, concentrations, membrane potentials and energy requirements resulting from changes in ion and water conductances, concentrations of permeant and impermeant ions, net valence of anions, and transporter and pump rates. Thus, it is quite general and can be used to investigate many situations. We have used it to investigate the influence of anions, cations and the transporters on cell volume and membrane potential. Some aspects of data obtained during our computational simulations have been discussed in the Results section. Here we will address three major points that deserve special attention.

### Na^+^/K^+^-ATPase is primarily responsible for the creation of the cation gradients and, consequently, the membrane potential, but does not directly participate in volume regulation

The first part of this statement brings no news, but the second does. Long ago the existence of a pump that actively extruded Na^+^ against its concentration gradient was postulated to explain cell volume [42, 43] and until now the key role of the Na^+^/K^+^-ATPase in volume regulation was not questioned [19, 23–25] (for review see [26]). Our simulation, however, demonstrated that in a system when only cations were concerned (and it is obvious that Na^+^/K^+^-ATPase deals only with cations) the pump is responsible for the voltage, but not for the volume. Any changes in the principal cation triumvirate - Na^+^ conductance, K^+^ conductance, Na^+^/K^+^-pump activity - always and inevitably lead to changes in *E*_*m*_ (Figs 4 and 5), even in theoretical conditions specifically designed to make an equal exchange of Na^+^ for K^+^ (electrically neutral 3Na^+^/3K^+^-pump, *gNa* = *gK*). But, as was well known, the cell voltage is much more sensitive to transmembrane movement of ions than the cell volume. The same amount of ions that is sufficient to charge the membrane capacitance and create a considerable change in *E*_*m*_ is negligibly small for cell volume and is associated with practically undetectable volume changes (Fig 5F). As a result, the slightest imbalance in total cation transfer across the membrane, which is irrelevant for the cell volume, can be important for *Em* and will stimulate strong negative feedback to prevent further imbalance. In this respect *Em* ensures osmotically balanced changes in Na^+^ and K^+^, and this is true in all conditions with any combination of the stoichiometry of the Na^+^/K^+^-ATPase, its activity, and cation conductances, including the case when the pump only removes Na^+^ from the cell (Fig 5C and 5D). Thus, the cations are not directly involved in cell volume regulation. It would be reasonable to say that Na^+^ and K^+^ are not for volume, but for voltage. Importantly, they have to pay for this privilege with ATP.

The ability of our program to show the definite ATP cost of the cation nonequilibrium appears to be useful for better understanding of relationships between ions, voltage and volume. For instance, the electrogenic pump needs more energy than our hypothetical electroneutral one to create the same concentration gradients. But the reason for the increased energy requirement is the different quantity of transferred ions per one cycle of the pump, not electrogenicity as is intuitively expected. To equilibrate [Na^+^]_i_ and [K^+^]_i_ (both equal to 75 mM, under the condition where *gNa* = *gK*), a 3Na^+^/2K^+^-pump which transfers 5 ions per cycle needs to spend 20% more ATP (179.8 million/sec) than a 3Na^+^/3K^+^-pump (149.8 million/sec) which transfer 6 ions per cycle. In both cases an equal number of cations is transferred per second (899 million) by the ATPase, and because these are resting states the same amount of ions passively leak back (Na^+^ into the cell, and K^+^ out of it). Of course, *Em* is more negative with an electrogenic pump than with an electroneutral one (-36.34 vs. -27.35 mV), but since a very small amount of ions produces this voltage shift, it is practically not reflected in the energy expenditure. The stoichiometry of the pump has a great influence on *Em*, and simulations, which are not shown, revealed that pumps that all transferred the same amount of charge per ATP, with Na^+^:K^+^ ratios of 6:0, 4:2, 3:3, 2:4 and 0:6 will generate -72.30, -42.34, -27.35, -12.37, and +17.60 mV of *Em*, respectively, in order to achieve [Na^+^]_i_ = [K^+^]_i_, but they all spend the same amount of energy with a precision of less than 0.002%. Thus, the energy is spent for cation electro-chemical gradients, which of course influence *Em*, but not for voltage itself via electrogenicity. Half the energy would be enough to reach this resting stage if the cell had half the cation conductance (S3 Fig). Interestingly, a cell can save a lot of energy supporting the cation electrochemical gradients and negative *Em* if its membrane is preferentially permeable to K^+^ (Fig 5C). The same electrogenic 3Na^+^/2K^+^-pump in the cell with the same total cation conductance needs 3.2 times less energy to equilibrate [Na^+^]_i_ and [K^+^]_i_ if *gK*/*gNa* = 9 compared to *gK*/*gNa* = 1 (55.8 million/sec instead of 179.8 million/sec). This is because the dominance of *gK* over *gNa* results in a smaller leak of the cations in spite of a much more negative *Em* (-66.11 vs. -36.34 mV).

It should be remembered that the importance of creating the transmembrane cation electro-chemical gradients by the Na^+^/K^+^-ATPase goes far beyond of generation of *Em*. These gradients (particularly the strong Na^+^ gradient) are heavily utilized by a cell for transport of metabolites, supporting Ca^2+^ homeostasis, controlling pH, and clearing neurotransmitters from the extracellular space, among other functions. These gradients can be used to cause non-equilibrium transmembrane distribution of Cl^-^, with all the following consequences. Changes in *Em* associated with changes in activity of the Na^+^/K^+^-ATPase (as well as changes in Na^+^ and K^+^ conductances) are also a prerequisite for possible cell volume changes. But in an “only cation system” all ionic transmembrane movements are osmotically balanced to satisfy macro electroneutrality. It is the addition of a membrane permeant anion (Cl^-^) what makes possible electrically neutral and osmotically significant ionic fluxes that lead to cell volume changes.

### The main factor that controls intracellular Cl^-^ concentration is the membrane potential, but it is Cl^-^ conductance that determines the extent of volume changes during normal neuronal activity

In the absence of specialized cotransporters (mainly the cation-Cl^-^ transporters that were modeled here and to some extent the bicarbonate-Cl^-^ transporter, which was beyond the scope of this work) Cl^-^ is distributed passively across the cell membrane. This means that Cl^-^ has to adjust its intracellular concentration to be in equilibrium with the cation-controlled *Em* (Fig 6A). When *Em* changes due to changes in cation conductance or Na^+^/K^+^-ATPase activity, [Cl^-^]_i_ is forced to change also in order to fit the new *Em*. How fast these changes in [Cl^-^]_i_ occur depends on the magnitude of the Cl^-^ conductance (Fig 7). If *gCl* is low, [Cl^-^]_i_ changes will develop slowly and will not be noticeable in a short time. But if *gCl* is high, [Cl^-^]_i_ changes will be comparable to those of [Na^+^]_i_ and [K^+^]_i_. Since Cl^-^ “shares the room” with impermeant intracellular anions (An^-^), all changes in [Cl^-^]_i_ must be associated with opposite sign changes in [An^-^]_i_ which is only possible if the cell volume is changed. Thus, alterations of *Em* induced by changes in cation (mostly Na^+^) conductance during normal neuronal activity will inevitably be accompanied by volume changes if *gCl* is substantial, or will have no visible volume effects, if *gCl* is low.

The presence of cation-Cl^-^ cotransporters complicates the matter. NKCC elevates [Cl^-^]_i_ above equilibrium and KCC lowers it below equilibrium. A nonequilibrium distribution of Cl^-^ enables this anion to contribute to *Em* (Fig 8). Regulation of Na^+^ conductance is still by far the more common (and more effective) way to manipulate *E*_*m*_ of neurons, because Na^+^ is much further from equilibrium than Cl^-^, but the contribution of unequally distributed Cl^-^ to *Em* should not be ignored (for review see [37]). For instance, *gCl* in combination with a nonequilibrium distribution of Cl^-^ plays an important role in such complex neuronal process as direction selectivity in the retina [29].

Still, the cation-Cl^-^ cotransporters do not disrupt the link between Cl^-^ and the cell volume. The cotransporters determine not the absolute value of [Cl^-^]_i_, but the Cl^-^ electro-chemical driving force, i.e. the difference from the concentration that would be equilibrium at current *Em*. When *Em* changes due to changes in *gNa*, *gK*, or the pump activities, [Cl^-^]_i_ is forced to adjust accordingly, leading to cell volume changes, just as in the case with no cation-Cl^-^ cotransporters.

To conclude this part, Cl^-^ may or may not influence *Em*, depending on its transmembrane distribution (nonequilibrium or equilibrium). But the presence of substantial *gCl* is absolutely necessary for activity-dependent cell volume changes. The importance of Cl^-^ in cell volume regulation was discussed theoretically and demonstrated experimentally in the literature (recently [46, 47]). What is stressed in this paper is the fact that *gCl*, not Na^+^/K^+^-ATPase, is responsible for volume changes. The apparent dependence of cell volume on the activity of the Na^+^/K^+^- ATPase can be explained by the following chain of events: changes in the activity of Na^+^/K^+^-ATPase lead to osmotically balanced (and therefore volume-irrelevant) changes in [Na^+^]_i_ and [K^+^]_i_, that in turn affect *E*_*m*_. With the presence of significant *gCl*, the changes in *Em* force [Cl^-^]_i_ to adjust accordingly. The transmembrane flux of Cl^-^ is electrically neutralized by a co-directed flux of cations and the resulting transfer of NaCl and KCl is osmotically noteworthy, leading to changes of the cell volume. So, swelling of the cell following suppression of the Na^+^/K^+^-ATPase could be avoided if it would be possible to completely block *gCl*.

### The concentration of the internal impermeant anions and their mean valence are major factors to determine volume but are limited contributors to voltage

Cl^-^ is “sharing room” with impermeant anion, An^-^, and the sum of [Cl^-^]_i_ and [An^-^]_i_ must be constant if extracellular concentrations remain unchanged. This undisputable fact has led to the suggestion that changes in [An^-^]_i_ should induce compensatory changes in [Cl^-^]_i_ and, consequently, that [An^-^]_i_ can be the key factor to determine [Cl^-^]_i_, making possible a nonequilibrium distribution of Cl^-^ [48]. This work was criticized from both theoretical [49] and experimental [50] points of view. And actually it also was shown computationally ten years earlier that a slow leak of An^-^ out of the cell diminishes the cell volume, but eventually does not change [Cl^-^]_i_ nor does it change [Na^+^]_i_, [K^+^]_i_, *E*_*m*_, and [An^-^]_i_ itself [28]. The same results were obtained during influx of An^-^ [25] as well as a buildup of An^-^ in this work (Fig 10C and 10D); only cell volume in those two cases increased because of An^-^ addition. Also, when [Cl^-^]_i_ was changed due to, for instance, the activity of cation-Cl^-^ cotransporters, the problem of keeping the sum of [Cl^-^]_i_ and [An^-^]_i_ constant was resolved by appropriate adjustment of “the room,” i.e. the cell volume (Fig 8).

However, when the mean valence of impermeant anions was altered, not only [Cl^-^]_i_, but also cation concentrations and *Em* are changed [25]. [Na^+^]_i_, [K^+^]_i_, [An^-^]_i_, [Cl^-^]_i_ and *Em* were also changed in our simulation of intracellular buildup of the neutral osmolyte (Fig 10A and 10B), which could be viewed as analogous to a decrease of mean valance of the impermeant anion (if the impermeant anion were defined as everything inside the cell except Na^+^, K^+^, and Cl^-^). In this work neutral osmolyte is treated separately from other internal impermeants, and they all are considered to be parts of a broader concept—the osmolarity-charge asymmetry.

The osmolarity-charge asymmetry inevitably arises when the internal impermeant anion (An^-^) has a mean valence (*z*) different from -1. The equation for internal macro electroneutrality is:

$${\left[K^{+}\right]}_{i}+{\left[Na^{+}\right]}_{i}=‑z*{\left[An^{‑}\right]}_{i}+{\left[Cl^{‑}\right]}_{i},$$ (37)

The complication here is that An^-^ is not a certain anion or even a set of anions of the same kind, but rather a collection of very different small and large molecules. In a cell with a membrane that is permeable only to Na^+^, K^+^ and Cl^-^, An^-^ can be defined as “everything which is internal, charged and impermeable” and it definitely must be an “anion” to compensate for the deficiency of negative charge of the main inorganic ions inside the cell. In this case the mean valence of An^-^ (*z*) is the ratio of all electrical charges that belong to An^-^ to the concentration of An^-^. Osmotically active proteins that carry multiple negative charges per molecule support the case for *z* < -1 (i.e. larger negative charge), but they are responsible for less than 10% of total cytoplasmic osmolarity [51]. Immobile proteins incorporated in the cell membranes, which can represent half of all proteins [52] provide some more negative charge without any osmotic contribution. However, the majority of [An^-^] is made of small organic molecules [53] that are mostly monovalent under physiological pH, such as creatine phosphate (about 40 mM in frog muscle [54]) and other phosphates and sulfates. Also, a significant part of the internal osmolytes is comprised of amino acids (up to 37 mM in rat brain, [55]), but among them only glutamate and aspartate are negatively charged, and some others are positively charged. Taking into account the wide diversity of components from which An^-^ is comprised, it is not surprising that the mean valence of An^-^ could be very different from cell to cell. In the case of myocytes a reasonable value of *z* is -1.65 [28], while for lymphoid cells it can be as large as -2.5 [23, 56]. Dusterwald and coworkers have used z = -0.85 [25].

Whatever *z* is, as long as *z* ≠ -1, it creates an asymmetrical osmolarity-charge setting, when the quantity of internal anions is not equal to the quantity of internal cations in an osmotic sense. The presence of internal neutral osmolytes, such as the just mentioned neutral amino acids, contributes to the asymmetry. A special place among them is occupied by sulfonic amino acid taurine, a zwitterion which is neutral at physiological pH. The concentration of taurine can be as high as 60 mM or even more, but varies significantly from species to species and from cell type to cell type, with higher concentration in mammals than in amphibians or reptiles, in retina than in brain or muscle, and in photoreceptors than in other retinal cells [57–59]. Addition of an external neutral osmolyte (although in the case of neural systems it is only a few mM, mostly from glucose) completes the equation of osmotic balance:

$${\left[K^{+}\right]}_{o}+{\left[Na^{+}\right]}_{o}+{\left[Cl^{‑}\right]}_{o}+{\left[osm\right]}_{o}={\left[K^{+}\right]}_{i}+{\left[Na^{+}\right]}_{i}+{\left[An^{‑}\right]}_{i}+{\left[Cl^{‑}\right]}_{i}+{\left[osm\right]}_{i}$$ (38)

where [osm] is the concentration of uncharged osmolytes.

To quantify the extent of the osmolarity-charge asymmetry a new parameter - the coefficient of asymmetry (*k*_*a*_) - is introduced as follows:

$$k_{a}=\left(‑z*{\left[An^{‑}\right]}_{i}+{\left[Cl^{‑}\right]}_{i}\right)/\left({\left[An^{‑}\right]}_{i}+{\left[Cl^{‑}\right]}_{i}+{\left[osm\right]}_{i}–{\left[osm\right]}_{o}\right)$$ (39)

Because charge of monovalent Na^+^ and K^+^ is equal to their osmolarity, both intracellular and extracellular, the charge-osmolarity imbalance, quantified by *k*_*a*_, results from anions and uncharged species. Thus, *k*_*a*_ is the ratio of all intracellular negative electrical charges to all osmotically active intracellular molecules except cations. The equation also includes external osmolyte, because addition of [osm]_o_ is the same for net osmolarity as subtraction of an equal amount of [osm]_i_. It is convenient to replace [osm]_i_−[osm]_o_ with d[osm], which is the difference in concentrations of internal and external neutral osmolytes and can be positive or negative. Accordingly, the equation for internal macro electroneutrality (Eq 37) can be rewritten in terms of *k*_*a*_:

$${\left[K^{+}\right]}_{i}+{\left[Na^{+}\right]}_{i}=k_{a}*\left({\left[An^{‑}\right]}_{i}+{\left[Cl^{‑}\right]}_{i}+d\left[osm\right]\right)$$ (40)

Since [K^+^]_o_ + [Na^+^]_o_ = [Cl^-^]_o_, Eq 38 for osmotic balance can be rewritten with regard to cations as:

$$2*{\left[cat\right]}_{o}={\left[cat\right]}_{i}+{\left[cat\right]}_{i}/k_{a}$$ (41)

where [cat]_i_ and [cat]_o_ are total intracellular and extracellular concentrations of the cations. From here one can derive the equation of osmolarity-charge asymmetry that describes the uneven, yet equilibrium cation distribution:

$${\left[cat\right]}_{i}/{\left[cat\right]}_{o}=2\;*k_{a}/\left(k_{a}+1\right);$$ (42)

Accordingly, *Em* in this equilibrium state with no Na^+^/K^+^-ATPase activity will be determine by *k*_*a*_:

$$Em=\left(RT/F\right)\;*\;ln\left(2\;*k_{a}/\left(k_{a}+1\right)\right)$$ (43)

In this condition, both *E*_*Na*_ and *E*_*K*_ must be equal to *Em*, and both cations separately follow Eq 42:

$${\left[Na^{+}\right]}_{i}/{\left[Na^{+}\right]}_{o}=2\;*k_{a}/\left(k_{a}+1\right);$$ (44)

$${\left[K^{+}\right]}_{i}/{\left[K^{+}\right]}_{o}=2\;*k_{a}/\left(k_{a}+1\right);$$ (45)

**Fig 11:**
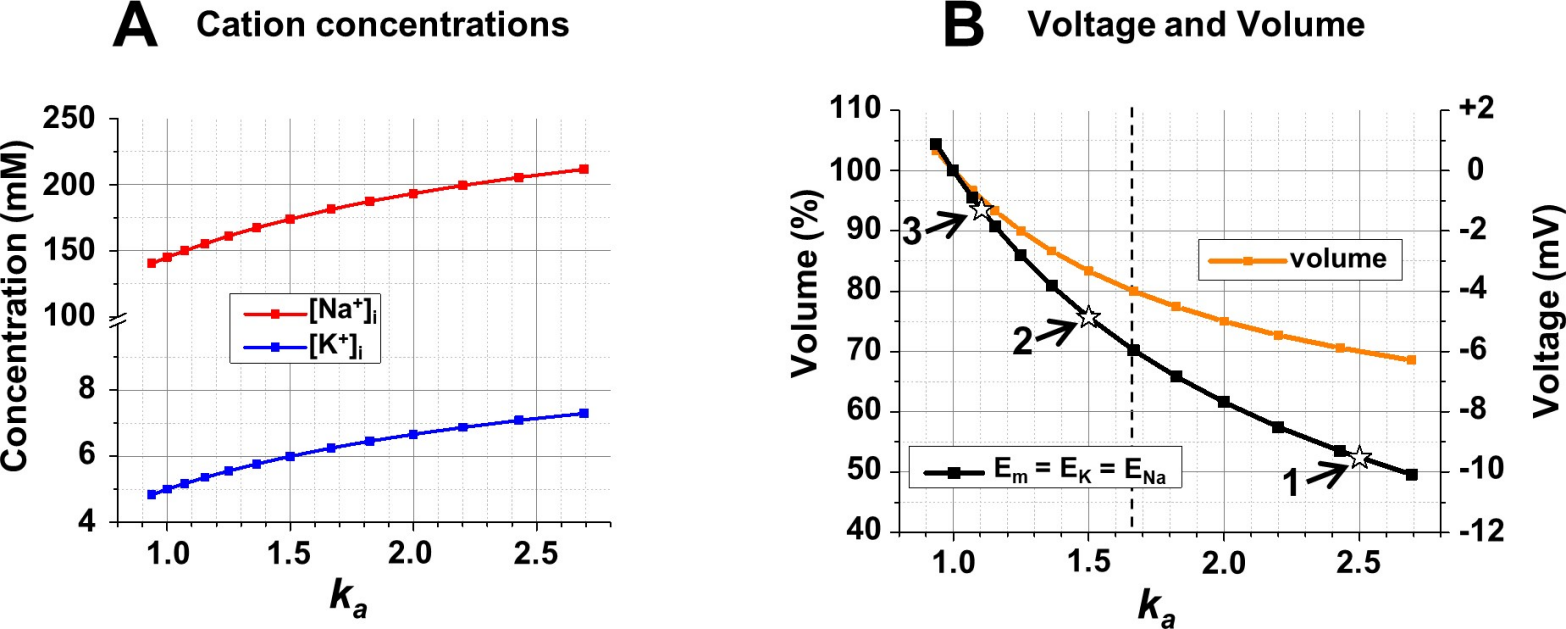
**The intracellular ionic concentrations (A), *E***_***m***_ **and the cell volume (B) as function of coefficient of asymmetry, *k***_***a***_**. A**: [Na^+^]_i_, and [K^+^]_i_ as a function of *k*_*a*_. Note the different scale for concentrations before and after the break. **B**: The cell volume (scale on the left), and *E*_*m*_, *E*_*K*_, and *E*_*Na*_ (scale on the right) as a function of *k*_*a*_. All three potentials have exactly the same values. The vertical dashed line marks the point when [osm]_i_ = [osm]_o_ = 0. The stars with numbers mark the data from previous simulations: 1 –as in Fig 4E, 2 –as in Fig 5B, 3 –as in Fig 4F. No Na^+^/K^+^-ATPase activity. **Initial conditions:** [Na^+^]_o_ = [Na^+^]_i_ = 145 mM; [K^+^]_o_ = [K^+^]_i_ = 5 mM; [Cl^-^]_o_ = 150 mM; [Cl^-^]_i_ = 30 mM; [An^-^]_i_ = 60 mM; z = -2.0; [osm]_i_ = 60 mM, which results in *k*_*a*_ = 1. The asymmetry with various *k*_*a*_ was created by addition of 10 mM of internal neutral osmolyte or reducing it down to 0 with 10 mM decrements, and then by increasing [osm]_o_ up to 50 mM with 10 mM increments. Note that the mean valence of impermeant anion (z) remains the same. Changes in osmolarity induced transmembrane water transfer that changed cell volume and intracellular concentrations.

This is true equilibrium when the concentration driving forces for both Na^+^ and K^+^ are countered by the electrical driving force, and osmotic balance also holds. In this respect it is similar to Double Donnan equilibrium, but with one important difference - the equilibrium based on the osmolarity-charge asymmetry is possible only if the cell membrane is permeable to cations, but not to Cl^-^. Opening of *gCl* will lead to inevitable and unlimited swelling. Of course, the system can be stabilized if the cation gradients are supported by the Na^+^/K^+^-ATPase. Thus, there are two factors of different nature that determine cation concentrations: one active, dependent on the cation conductances and the pump activity, and the other passive, dependent on *k*_*a*_. Accordingly, there are two parts of cation-dependent *Em*. The active part of *Em* is much larger than the passive (Fig 5E), and also the active part can be quickly, in a small fraction of a second, changed by manipulating cation conductances. There is no doubt that this active component, which is determined by *gNa*, *gK* and the activity of the Na^+^/K^+^-ATPase, dominates *Em*. On other hand, although the passive osmolarity-charge asymmetry dependent component is probably present in *Em* of every cell (since it is very unlikely that *k*_*a*_ = 1 in any cell), its contribution will be insignificant in most conditions. Changes in *k*_*a*_, are probably common since they can be a result of changes in z, [An^-^]_i_, [osm]_i_, or [osm]_o_, but in most cases with small effect on *Em*. As illustrated in Fig 11B, a decrease of *k*_*a*_ from 2 to 1.5 (which was achieved by an increase of d[osm] by 32 mM with constant *z*) depolarized *Em* by only 2.8 mV, since it was associated with relatively insignificant changes in [K^+^]_i_ and [Na^+^]_i_ (from 6.9 to 6.0 mM and from 193.3 to174 mM, respectively, Fig 11A). Also accompanying this was a modest 8.3% increase in cell volume (normalized to the volume in osmolarity-charge *symmetric* conditions when *k*_*a*_ = 1). The small changes in *Em* (about 2 mV) demonstrated in simulations of osmotic disturbances (Fig 9A and 9B, Fig 10A and 10B) are a consequence of changes in osmolarity-charge asymmetry, when the cell was slightly hyperpolarized due to an increase of *k*_*a*_ resulting from the buildup of external osmolyte and slightly depolarized due to a decrease of *k*_*a*_ resulting from the buildup of internal osmolyte. But when *Em* was changed, [Cl^-^]_i_ had to change too, assuming the presence of substantial *gCl*. In this respect Glykys and coauthors [48] were right in claiming that impermeant anion An^-^ can influence [Cl^-^]_i_, although it happened not because of a direct link, but due to a chain of events including changes in osmolarity-charge asymmetry, cation concentrations and *Em*. However, if Cl^-^ was in equilibrium with *Em*, it will continue to be in equilibrium.

Importantly, osmolarity-charge asymmetry is also affected by changes in [Cl^-^]_i_. if the distribution of ions was already asymmetric. As was mentioned in the results concerning Fig 8E, the elevation of [Cl^-^]_i_ by NKCC in “realistic” (i.e. osmolarity-charge asymmetric) conditions led to a decrease of the total internal anion charge because An^-^ with z = -1.5 was replaced by the monovalent Cl^-^. The inevitable result of that were changes of cation concentrations and *Em*. Effects of cation-Cl^-^ cotransporter-induced changes in [Cl^-^]_i_ on [Na^+^]_i_, [K^+^]_i_, and *Em* can be revealed by comparing “realistic” conditions with “simplified” (i.e. osmolarity-charge *symmetric*) conditions, especially when *gCl* was low (10^8^ ions/(sec*V)) and Cl^-^ practically had no direct contribution to *Em*. When Cl^-^ was pumped by cation-Cl^-^ cotransporters in symmetric “simplified” conditions, it replaced (or was replaced by) an equal quantity of monovalent An^-^. As a result, *k*_*a*_ continues to be 1, [Na^+^]_i_ and [K^+^]_i_ remain almost the same, and slight changes of *Em* did not exceed 0.3 mV (Fig 8A for NKCC and Fig 8C for KCC). But in asymmetric “realistic” conditions an increase of [Cl^-^]_i_ by NKCC resulted in a reduction of *k*_*a*_ from 1.47 to 1.28, a decrease of [Na^+^]_i_ and [K^+^]_i_ and a depolarization by 1.42 mV (see S4A Fig); a decrease of [Cl^-^]_i_ by KCC led to smaller changes in *k*_*a*_ (from 1.47 to 1.56) and a subsequent increase in both cation concentrations and hyperpolarization by -0.78 mV (S4B Fig).

Finally, it should be noted that this asymmetry-dependent voltage is completely determined by *k*_*a*_ and is independent of the internal ionic and osmotic compositions as long as they result in the same *k*_*a*_. The data for Fig 11 were obtained during manipulation of the neutral osmolytes (see explanation in the figure legend), but the stars with numbers were from our previous simulations with different internal compositions (star 1: Fig 4E, star 3: Fig 4F, and star 2: Fig 5B, “realistic” conditions; all for the points at the left of the graphs where there is no Na/K pumping). The results of computational simulations were exactly the same as predictions from Eqs 43, 44, and 45.

To summarize, [An^-^]_i_ and its mean valence play an important role in determination of cell volume. It was shown earlier, and it was confirmed here. [An^-^]_i_ and its mean valence also, together with other factors ([Cl^-^]_i_, internal and external neutral osmolytes), contribute to creation of osmolarity-charge asymmetry, which passively influence cation distribution and *Em*, although the effect is small compared to the active Na^+^/K^+^-ATPase dependent cation voltage.

## Supporting information

S1 Text On/Off excitation in vertebrate retina. (DOCX)

S2 Text Shunting inhibition. (DOCX)

S1 Fig Initial voltage changes after opening of Na^+^ and Cl^-^ conductances. With *gNa* = *gCl*, *E*_*m*_ after opening of *gNa* and *gCl* must be half way between of *E*_*Na*_ and *E*_*Cl*_, which is -30.74 mV (*E*_*Na*_ = 0, *E*_*Cl*_ = -61.48mV). It takes about 25 ms of exponential changes of *E*_*m*_ to achieve this level. The characteristic point of 63.21% (1-1/*e*) of maximal amplitude (Amax) was reached in 3.7 ms. This fits the calculated time constant of our modeled cell (RC = 3.75*10^−3^ s, since R = 3.125*10^8^ Ω and C = 1.2*10^−11^ F) with good precision, remembering that the time step in this calculation was 0.1 ms. Additionally, this figure illustrates the huge difference in time scales of voltage and concentration changes. Only 25 ms was enough to achieve *electrical equilibrium* of *E*_*m*_ with respect to *E*_*Na*_ and *E*_*Cl*_, but it took about 25 minutes (60000 times longer) to equilibrate concentrations (Fig 1A).
(TIF)

S2 Fig Disruption of Double Donnan equilibrium by the presence of a small Na^+^ conductance. The conditions here were the same as in calculations shown in Fig 3C and 3D with one exception: a small *gNa* (10^8^ ions/sec*V, i.e. a bit less than 0.5% of total transmembrane conductance) was present. Initially (first 10 min, parts A and B), all changes were similar to Fig 3C and 3D, but importantly neither concentrations, nor volume, nor voltage stabilized. These parameters never reach equilibrium as illustrated by parts C and D of this figure, where the same changes are presented on longer time scale.
(TIF)

S3 Fig **Dependence of [Na**^**+**^**]**_**i**_**, [K**^**+**^**]**_**i**_ **(scale on the left), and *E***_***m***_ **(scale on the right) on activity of the electrically neutral (A) and electrogenic (B) 3Na**^**+**^**/2K**^**+**^**-pump.** The conditions are the same as in Fig 4B and 4C, respectively, but *gNa* and *gK* are half of those values in Fig 4 (both are 5*10^9^ instead of 10^10^ ions/s*V), and accordingly the cell input resistance is twice as large (625 instead of 312.5 MΩ). As a result, half of the energy was needed to achieve the same ionic gradients and *Em* as in the simulations presented in Fig 4B and 4C.
(TIF)

S4 Fig The intracellular ionic concentrations, *E*_*m*_ and the cell volume as a function of the activity of cation-Cl^-^ cotransporters. NKCC (A) and KCC (B) work against low *gCl* (10^8^ ions/(sec*V)), both in “realistic” conditions; all other parameters and axes are the same as in Fig 8A and 8C.
(TIF)
