# Supplementary material for: The logic of ionic homeostasis: Cations are for voltage, but not for volume
Source: PLoS Comput Biol. 2019 Mar 14;15(3):e1006894. doi: 10.1371/journal.pcbi.1006894 (PMC6435201; doi:10.1371/journal.pcbi.1006894)
Supplement: S1 Text — (DOCX) [file pcbi.1006894.s001.docx]

**On/Off Excitation in vertebrate retina.** Existence of On- and Off-pathways in the vertebrate retina implies that some postreceptor neurons will respond with depolarization and others with hyperpolarization at both onset and offset of the light stimulus. However, depolarization dominates in proximal retina due to nonlinearities in the signal processing. As proof of this, [K^+^]_o_ always increases in proximal retina in response to both onset and offset of light in all vertebrate retinae tested.
