# Supplementary material for: The logic of ionic homeostasis: Cations are for voltage, but not for volume
Source: PLoS Comput Biol. 2019 Mar 14;15(3):e1006894. doi: 10.1371/journal.pcbi.1006894 (PMC6435201; doi:10.1371/journal.pcbi.1006894)
Supplement: S2 Text — (DOCX) [file pcbi.1006894.s002.docx]

**Shunting inhibition.** Shunting inhibition is a way to explain diminishing of cation-induced excitation by Cl^-^-carrying channels from the point of view of the Ohm’s law. Cl^-^ could be in equilibrium and would not affect *Em* directly, but Cl^-^ conductance still contributes to total transmembrane conductance, decreasing the cell input resistance. The larger the Cl^-^ conductance, the smaller will be transmembrane resistance, and the smaller the voltage changes (usually depolarizations) associated with increases in cation (predominantly Na^+^) conductances. From the charge-capacitance perspective, Na^+^-dependent depolarization disturbs Cl^-^ electro-chemical balance and stimulates Cl^-^ influx. As a result, Cl^-^ partly neutralizes internal positive charge and diminishes Na^+^-induced depolarization.
