# Supplementary figures and images for: The logic of ionic homeostasis: Cations are for voltage, but not for volume
Source: PLoS Comput Biol. 2019 Mar 14;15(3):e1006894. doi: 10.1371/journal.pcbi.1006894 (PMC6435201; doi:10.1371/journal.pcbi.1006894)

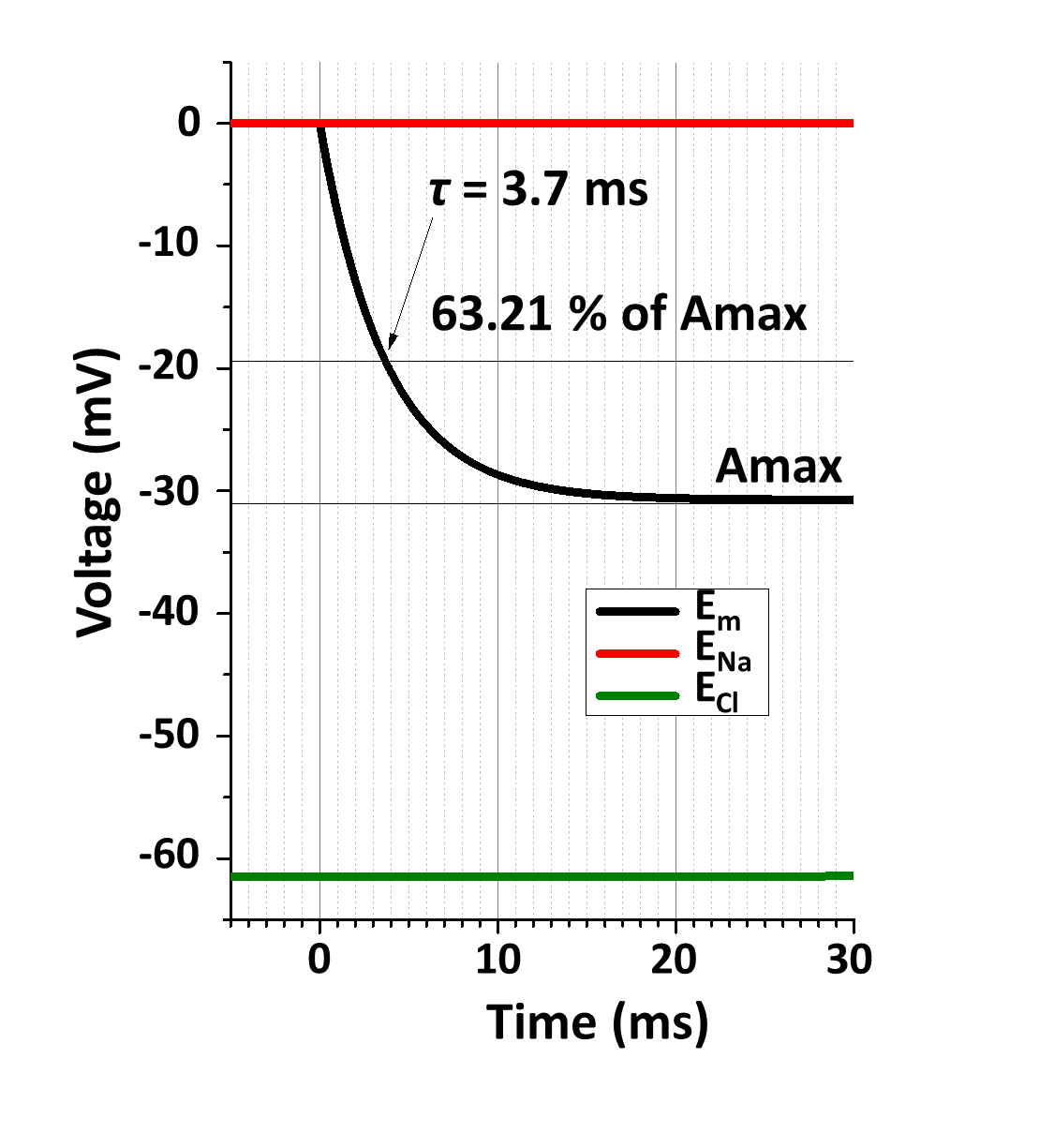

Supplement: S1 Fig — With gNa = gCl, Em after opening of gNa and gCl must be half way between of ENa and ECl, which is -30.74 mV (ENa = 0, ECl = -61.48mV). It takes about 25 ms of exponential changes of Em to achieve this level. The characteristic point of 63.21% (1-1/e) of maximal amplitude (Amax) was reached in 3.7 ms. This fits the calculated time constant of our modeled cell (RC = 3.75*10−3 s, since R = 3.125*108 Ω and C = 1.2*10−11 F) with good precision, remembering that the time step in this calculation was 0.1 ms. Additionally, this figure illustrates the huge difference in time scales of voltage and concentration changes. Only 25 ms was enough to achieve electrical equilibrium of Em with respect to ENa and ECl, but it took about 25 minutes (60000 times longer) to equilibrate concentrations (Fig 1A). (TIF) [file pcbi.1006894.s003.tif]

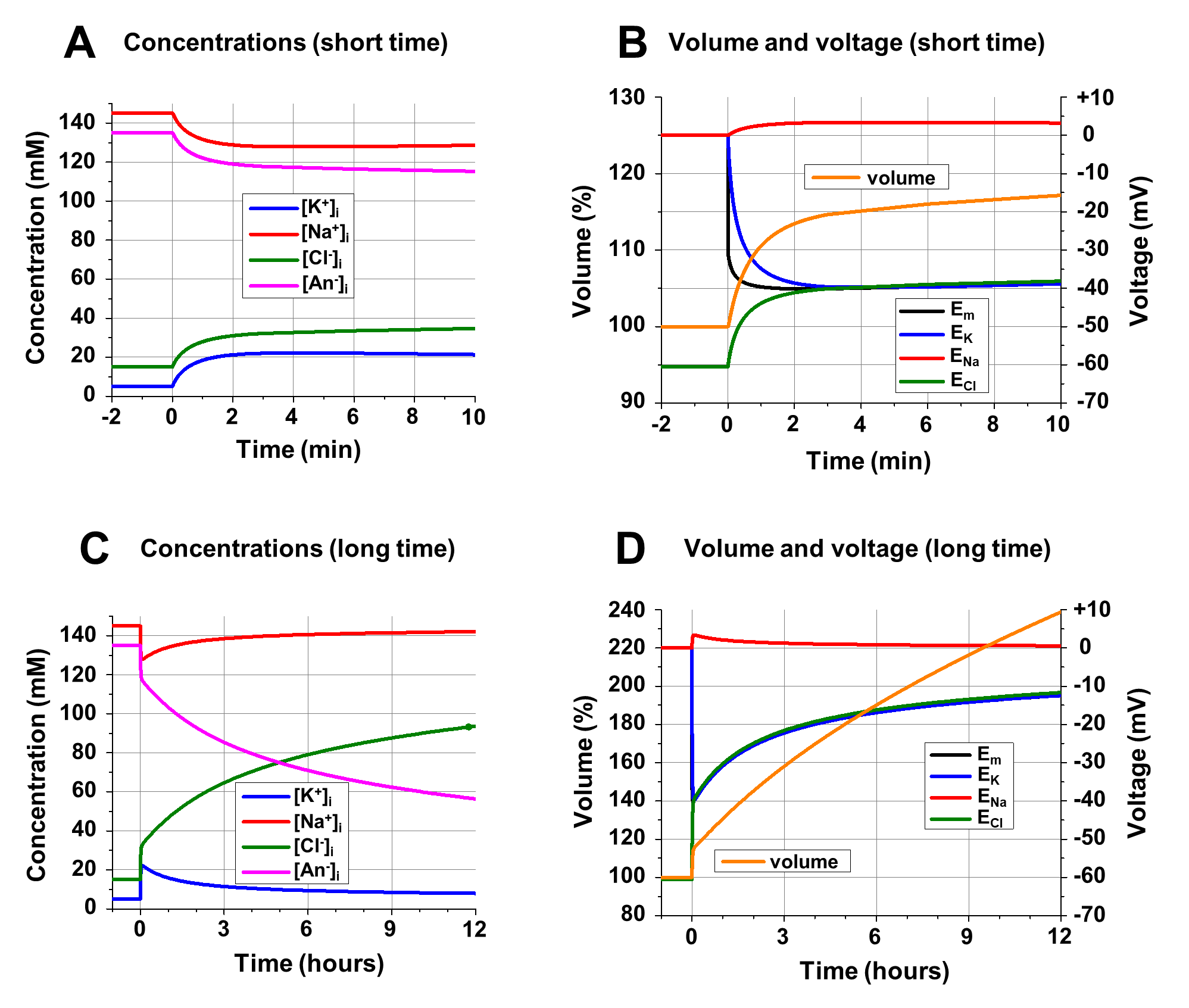

Supplement: S2 Fig — The conditions here were the same as in calculations shown in Fig 3C and 3D with one exception: a small gNa (108 ions/sec*V, i.e. a bit less than 0.5% of total transmembrane conductance) was present. Initially (first 10 min, parts A and B), all changes were similar to Fig 3C and 3D, but importantly neither concentrations, nor volume, nor voltage stabilized. These parameters never reach equilibrium as illustrated by parts C and D of this figure, where the same changes are presented on longer time scale. (TIF) [file pcbi.1006894.s004.tif]

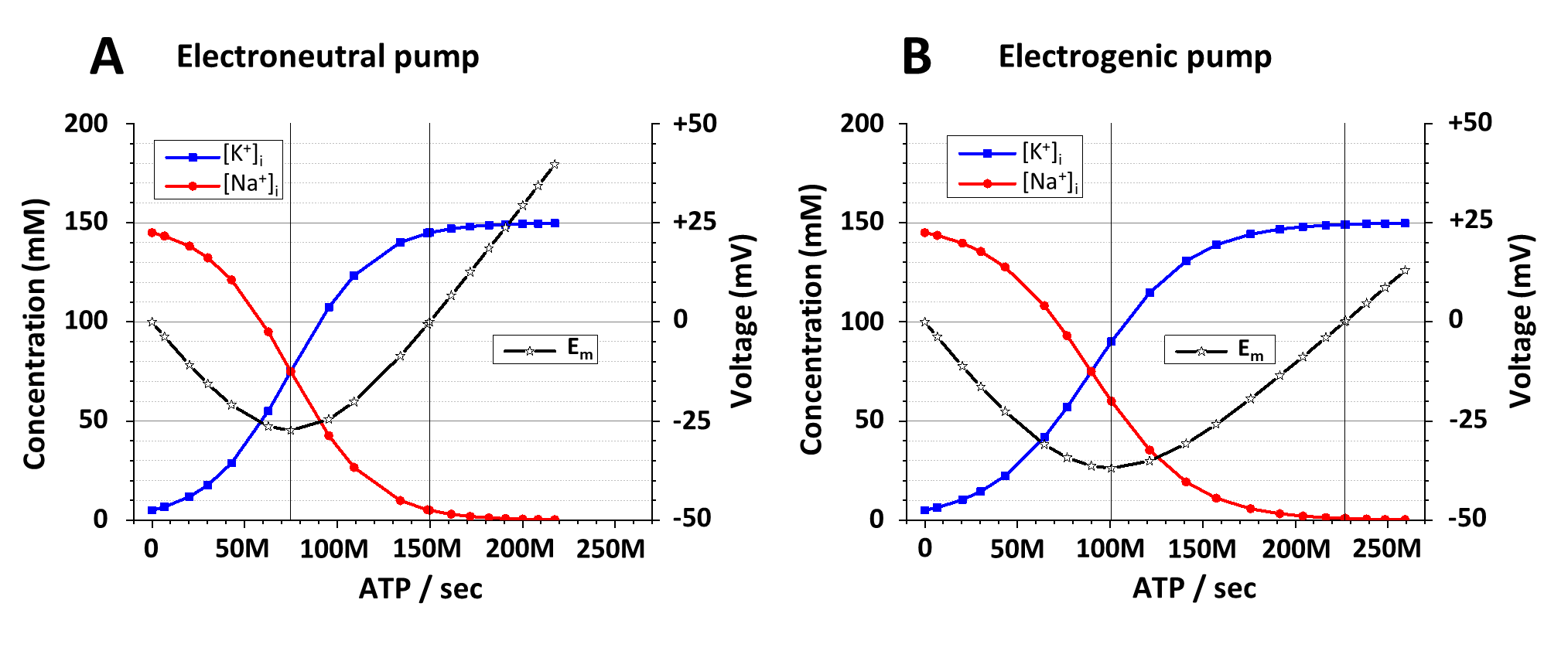

Supplement: S3 Fig — Dependence of [Na+]i, [K+]i (scale on the left), and Em (scale on the right) on activity of the electrically neutral (A) and electrogenic (B) 3Na+/2K+-pump. The conditions are the same as in Fig 4B and 4C, respectively, but gNa and gK are half of those values in Fig 4 (both are 5*109 instead of 1010 ions/s*V), and accordingly the cell input resistance is twice as large (625 instead of 312.5 MΩ). As a result, half of the energy was needed to achieve the same ionic gradients and Em as in the simulations presented in Fig 4B and 4C. (TIF) [file pcbi.1006894.s005.tif]

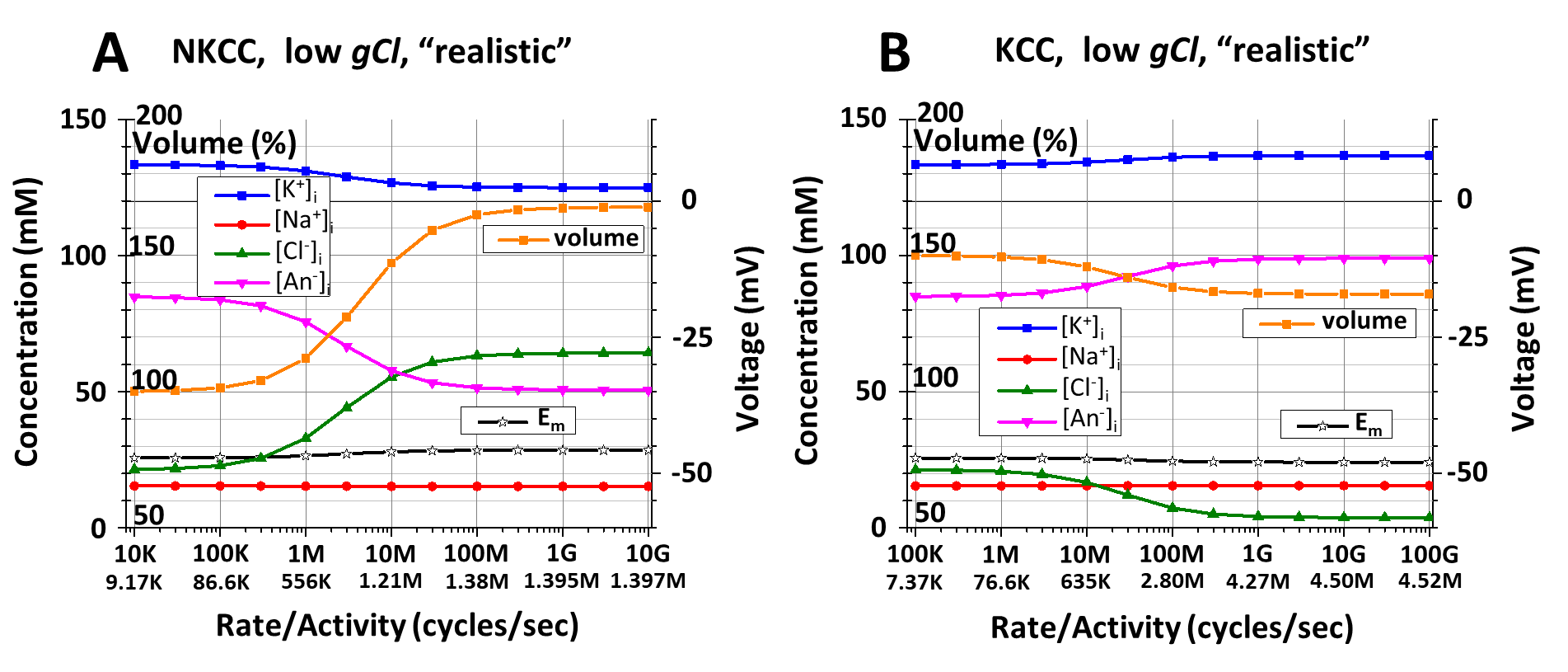

Supplement: S4 Fig — NKCC (A) and KCC (B) work against low gCl (108 ions/(sec*V)), both in “realistic” conditions; all other parameters and axes are the same as in Fig 8A and 8C. (TIF) [file pcbi.1006894.s006.tif]
